# Integrative analysis of spatial and single-cell transcriptome data from human pancreatic cancer reveals an intermediate cancer cell population associated with poor prognosis

**DOI:** 10.1186/s13073-024-01287-7

**Published:** 2024-01-31

**Authors:** Seongryong Kim, Galam Leem, Junjeong Choi, Yongjun Koh, Suho Lee, Sang-Hee Nam, Jin Su Kim, Chan Hee Park, Ho Kyoung Hwang, Kyoung Il Min, Jung Hyun Jo, Hee Seung Lee, Moon Jae Chung, Jeong Youp Park, Seung Woo Park, Si Young Song, Eui-Cheol Shin, Chang Moo Kang, Seungmin Bang, Jong-Eun Park

**Affiliations:** 1 Graduate School of Medical Science and Engineering, Korea Advanced Institute of Science and Technology, 291 Daehak-Ro, Yuseong-Gu, Daejeon, 34141 Republic of Korea; 2 Division of Gastroenterology, Department of Internal Medicine, Severance Hospital, Yonsei University College of Medicine, 50-1 Yonsei-Ro, Seodaemun-Gu, Seoul, 03722 Republic of Korea; 3 Department of Pharmacy and Yonsei Institute of Pharmaceutical Sciences, College of Pharmacy, Yonsei University, Incheon, Republic of Korea; 4 Department of Internal Medicine, Graduate School of Yonsei University, Seoul, Republic of Korea; 5 Division of Hepatobiliary and Pancreatic Surgery, Department of Surgery, Yonsei Cancer Center, Yonsei University College of Medicine, Pancreatobiliary Cancer Center, Severance Hospital, 50-1 Yonsei-Ro, Seodaemun-Gu, Seoul, 03722 Republic of Korea; 6 Pancreatobiliary Cancer Center, Yonsei Cancer Center, Severance Hospital, Seoul, Republic of Korea

**Keywords:** Pancreatic cancer, Pancreatic cancer cells, Transitional cell state, Molecular subtype of pancreatic cancer, Cancer-associated fibroblasts

## Abstract

**Background:**

Recent studies using single-cell transcriptomic analysis have reported several distinct clusters of neoplastic epithelial cells and cancer-associated fibroblasts in the pancreatic cancer tumor microenvironment. However, their molecular characteristics and biological significance have not been clearly elucidated due to intra- and inter-tumoral heterogeneity.

**Methods:**

We performed single-cell RNA sequencing using enriched non-immune cell populations from 17 pancreatic tumor tissues (16 pancreatic cancer and one high-grade dysplasia) and generated paired spatial transcriptomic data from seven patient samples.

**Results:**

We identified five distinct functional subclusters of pancreatic cancer cells and six distinct cancer-associated fibroblast subclusters. We deeply profiled their characteristics, and we found that these subclusters successfully deconvoluted most of the features suggested in bulk transcriptome analysis of pancreatic cancer. Among those subclusters, we identified a novel cancer cell subcluster, Ep_VGLL1, showing intermediate characteristics between the extremities of basal-like and classical dichotomy, despite its prognostic value. Molecular features of Ep_VGLL1 suggest its transitional properties between basal-like and classical subtypes, which is supported by spatial transcriptomic data.

**Conclusions:**

This integrative analysis not only provides a comprehensive landscape of pancreatic cancer and fibroblast population, but also suggests a novel insight to the dynamic states of pancreatic cancer cells and unveils potential therapeutic targets.

**Graphical Abstract:**

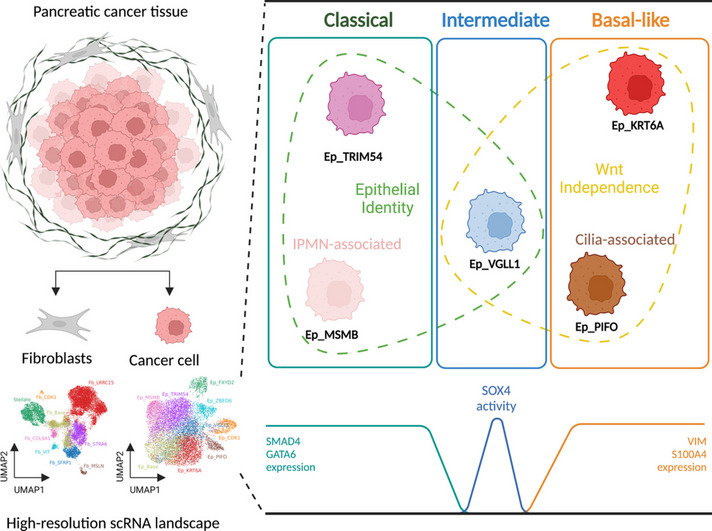

**Supplementary Information:**

The online version contains supplementary material available at 10.1186/s13073-024-01287-7.

## Background

Pancreatic cancer is currently the fourth leading cause of cancer-related death [1] and is expected to become the second leading cause by 2040 [2]. Despite advances in the diagnosis and treatment of cancer patients over the past few decades, the 5-year survival rate of pancreatic cancer patients is still under 10% [1], because over 80% of pancreatic cancer patients are diagnosed with unresectable or metastatic disease and receive systemic chemotherapy as first-line therapy. The currently preferred regimens, FOLFIRINOX or gemcitabine plus nab-paclitaxel (Abraxane), do not extend patient survival by more than 12 months [3–5]. To improve the clinical outcomes, a comprehensive understanding of the biological properties of pancreatic cancer cells and other cancer-associated cells is required.

Previous studies using bulk transcriptome analysis consistently reported two major molecular subtypes in pancreatic cancer: classical and basal-like (also identified as quasi-mesenchymal) subtypes [6–9]. This dichotomous classification is highly correlated with prognosis and responses to chemotherapies [6, 10]. Recently, several studies have performed single-cell RNA sequencing (scRNA-seq) to dissect the heterogeneous tumor microenvironment in pancreatic cancer tissues to define the molecular subtypes of pancreatic cancer [11–16] and reported co-existence of the classical and basal-like cell clusters in pancreatic cancer tissues. However, because of the high inter-patient heterogeneity of the tumor microenvironment and low cellularity of cancer cells, an integrative and comprehensive map of pancreatic cancer cells has not been clearly suggested yet [17].

Here, to investigate the landscape of epithelial cells and fibroblasts precisely and to analyze each subpopulation in an integrated manner, we deeply sequenced non-immune cells from various pancreatic tumor tissues: 13 pancreatic ductal adenocarcinoma (PDAC) tumor tissues and 4 intraductal papillary mucinous neoplasms (IPMNs) with high-grade dysplasia or invasive carcinoma tissues. We identified five distinct functional cancer cell clusters and six fibroblast clusters from the detailed map of single-cell transcriptome data. This integrative map of pancreatic cancer cells and cancer-associated fibroblasts successfully deconvoluted diverse aspects of pancreatic cancer. Furthermore, we identified a new cancer cell cluster, Ep_VGLL1, which shows intermediate characteristics between classical and basal subtypes, despite its prognostic implications.

## Methods

### Patient information and sample collection method

We prospectively enrolled patients who were newly diagnosed with pathologically confirmed pancreatic cancer between January 2019 and July 2020 at Severance Hospital (Seoul, Korea). A total of 17 patients were enrolled; 13 patients were diagnosed with PDAC and one patient with IPMN with high-grade dysplasia (HGD) and 3 patients with invasive carcinoma. All patients diagnosed with IPMN were diagnosed with side-branch duct IPMN. Additional file 1: Table S1 summarizes the clinical characteristics of the patients and Additional file 2: Fig. S1 shows representative histology of the patients. One patient with stage IV pancreatic cancer underwent surgery and was included in this study because the patient exhibited no signs of metastasis and was clinically diagnosed with T2N0M0 before the surgery. A cluster of atypical pancreatobiliary epithelial cells, suggesting peritoneal carcinomatosis, was confirmed via the peritoneal fluid cytology performed during the surgery; thus, the patient was pathologically diagnosed with T2N0M1 after the surgery. All patients provided written informed consent. The study was conducted in accordance with the Declaration of Helsinki (1996) and approved by the Institutional Review Board of Yonsei University Medical Center (number 4–2018-0780). The surgical tissues were collected consecutively from 17 patients who provided informed consent to participate in the study among patients who were diagnosed with pancreatic tumor and planned surgery during the study period.

We collected fresh tumor tissues, including tumor core lesion, via surgical resections of the 17 patients enrolled in this study. We obtained single-cell suspensions from the tumor tissues with enzymatic and mechanical digestion, as described previously [18]. Briefly, we cut fresh tissues into small pieces using dissection scissors (2–4 mm) and transferred the pieces to gentle MACS C-tubes (Miltenyi Biotec, Bergisch Gladbach, Germany) containing a mixture of enzymes (Enzyme H, R, and A from the human Tumor Dissociation Kit, Miltenyi Biotec). Transferred tissues were mechanically homogenized and enzymatically digested for 1 h using the gentle MACS Octo Dissociator (Miltenyi Biotec). After digestion, the cell suspensions were passed through 40-µm pore cell strainers and washed once. The cells were cryopreserved until use.

### Single-cell transcriptome data generation and analysis

#### Cell sorting

We isolated CD45-negative cells from the single-cell suspension obtained from fresh pancreatic cancer tumor tissues using magnetic-activated cell sorting (MACS; Miltenyi Biotec). After thawing the cryopreserved single-cell suspensions, we depleted cell debris, dead cells, and dying cells using the Dead Cell Removal Kit (Miltenyi Biotec). Next, we labeled CD45-positive cells in live single-cell suspensions using human CD45 MicroBeads (Miltenyi Biotec) and sorted the unlabeled CD45-negative cells by MACS. Only the flowthroughs proceeded to single-cell library construction. For three of the PDAC patient surgical samples, CD45-positive cells were also collected and processed with CD45-negative cells.

#### Single-cell RNA library construction and sequencing

We multiplexed the cells from three to four surgical samples by matching the cell counts and constructed single-cell RNA libraries with a target cell number of 10,000 to 15,000 per library using Chromium Next GEM Single Cell 5’ library v1.1 (10 × Genomics, Pleasanton, CA, USA) according to the manufacturer’s instructions. Each single-cell RNA library was sequenced using the NovaSeq 6000 Sequencing System (Illumina, San Diego, CA, USA) to obtain approximately 50,000 reads per cell.

#### Data preprocessing procedures

Single-cell RNA sequencing data were sequentially processed with the Cell Ranger pipeline [19] (v.4.0.0) with GRCh38 2020-A as the reference genome (GENCODE v32/Ensembl 98). Subsequent data analysis was performed in the Scanpy [20] (v1.8.2) package. Only the cells meeting the following criteria were included in the analysis: (1) UMI counts > 2000, (2) number of detected genes from 500 to 7000, (3) percentage of mitochondrial genes < 10%, and (4) Scrublet [21] (v0.2.2) predicted singlet. To demultiplex the samples in a single 10X scRNA-seq library, we utilized Souporcell [22] (v2.0) with *k* values of 3 or 4. To match the patient information with the Souporcell output, we compared the Souporcell output with the single-nucleotide variant information acquired from the patient PBMCs by SNP array kit (Infinium Asian Screening Array-24 v1.0).

Filtered count matrices were count-normalized, log-transformed, and processed using the following steps. First, we identified highly variable genes with default parameters in Scanpy, which yielded 2536 and 2267 highly variable genes for epithelial and fibroblast populations, respectively. We scaled the expression matrices and subsequently performed PCA (number of PCs = 50). The PCs were corrected (patient-wise) with Harmony (harmonypy v0.0.5) [23], and batch-balanced neighborhood graphs were constructed with BBKNN (v1.4.1) [24]. UMAP coordinates were computed based on the neighborhood graphs and we used Leiden clustering (resolution 2.0) for initial clustering of the cells.

### Marker gene selection

The marker gene candidates for each cluster were selected based on the specificity and average expression values per cluster were maximally normalized based on the top-expressing cluster (top-expressing cluster average value set to 1), with the aim that the gap between the top and second-highest clusters could represent specificity. All genes were then ordered based on the ranking scored by the gap value. To avoid selecting lowly expressed genes as marker genes, we also applied expression criteria (average log-normalized expression value of the top-expressing cluster was over 0.4, and the fraction of cells expressing the gene in the cluster was greater than 10%). Finally, we manually inspected the top-ranked marker gene candidates to ensure that they were consistently detected in different patients and then performed Wilcoxon rank-sum tests to generate the final version of marker genes.

### TCGA data analysis

Bulk RNA sequencing data reflect mixed transcriptomic features from various cell types in the tissue. Since we established the marker set based on the cluster-specific expression within each cell type, the markers do not guarantee expression specificity across all the other cell types in the tumor tissue. Thus, we manually filtered out the marker genes that are expressed in all the other major cell types in our scRNA-seq data and defined the curated version of the marker gene set as “refined marker gene set.” We calculated “subcluster scores” for each sample in the TCGA and ICGC cohorts by calculating the average expression of genes included in the refined marker gene sets.

To determine the optimal cut-off values for survival analysis, we first identified Q1 (25% percentile) and Q3 (75% percentile) values of the subcluster scores. Next, we defined potential cut-off values as every possible value between Q1 and Q3 by a margin of 0.04. Then, we split the “score-high” group and “score-low” group according to each potential cut-off value and tested whether the two groups are showing different survival patterns using a log-rank test implemented in lifelines (v0.26.4) package. For each subcluster score, the cut-off value with the lowest *P*-value was designated as an optimal cut-off value [25].

To address overfitting issues, we cross-validated the cut-off parameters. Briefly, we used standardized gene expression values from each dataset (TCGA and PACA-CA) and calculated the mean expression profiles of each cluster using the refined marker gene sets. Using the mean expression profiles and the cut-off determination strategies described above, we first acquired the optimal cut-off values for each cluster marker in the TCGA dataset. Subsequently, we split the patient samples in each cohort (TCGA and PACA-CA) according to the optimal cut-off values trained from the TCGA dataset and statistically evaluated whether the two groups in each cohort showed differential prognostic patterns with log-rank tests.

### RNA in situ hybridization

Tissue slides were obtained from FFPE (formalin-fixed and paraffin-embedded) blocks of pancreatic cancer tissues, adjacent to the tissue area used for single-cell RNA sequencing. The 5-µm-thick formaldehyde-fixed paraffin-embedded tissue sections were deparaffinized with Xylene and subsequently processed with RNA scope Multiplex Fluorescent Reagent Kit Assay. Transcripts in the slides were hybridized with RNAscope probes (VGLL1: AD44673, TRIM54: AD555211-C3, KRT6B: AD805641-C4, KRT19: AD310221-C2, PDGFRA: AD604481-C3, COCH: AD1104401-C1, TSLP: AD403541-C2, PI16: AD569181, CD34: AD560821-C2). Fluorescence signals were detected with Pannoramic SCAN II (3D Histech) using FITC (OPAL 520), TRITC (OPAL 570), Cy5.5 (OPAL 690), and DAPI channel.

### Immunohistochemistry

In this study, 5-µm-thick FFPE tissue sections were subjected to routine hematoxylin and eosin staining using Muto Pure chemical (2002–2). Immunohistochemistry was performed on FFPE tissue sections using an automatic immunohistochemical staining device (Benchmark XT, Ventana Medical System). The samples were treated with specific primary antibodies, including COL9A1 (LSBio, LS-C98645, 1:200), KRT6B (Abcam, ab154313, 1:500), SPRR3 (Bioss, BS-11163R, 1:200), and TRIM54 (Origene, TA803871, 1:150).

### Spatial transcriptomic data analysis

#### Spatial transcriptome library construction and sequencing

We obtained FFPE blocks of the tumor tissues, adjacent to the tissue area on which we performed scRNA-seq from seven patients with pancreatic cancer. We performed hematoxylin and eosin (H&E) staining and selected regions of interest to include an adequate number of cancer cells and stromal tissues by 6.0 × 6.0 mm. We then performed RNA probe hybridization, ligation, and barcoding to construct spatial transcriptomic libraries using Visium Spatial Gene Expression Reagent Kits for FFPE (10 × Genomics) according to the manufacturer’s instructions. Each FFPE library was sequenced using a NovaSeq 6000 Sequencing System (Illumina).

#### Spatial deconvolution using reference single-cell transcriptomic data

We first concatenated single-cell transcriptomic data from both CD45-negative and CD45-positive datasets, and the integrated dataset was used as the reference. The cellular abundance estimations for each spot in the spatial transcriptomic data were generated using a Bayesian inference-based spatial deconvolution tool Cell2location [26]. The 5% quantile estimates of cluster abundance in each spot were used as spatial abundance estimates in the downstream analysis.

#### Neighborhood enrichment analysis and graph construction

We first identified the “high spots” for each cell type using abundance profiles projected by Cell2location (spots with 5% quantile abundances > 3 were defined as the high spots). For each high spot, the abundance profiles of neighboring spots (the spots up to the third most proximal spots) were summed. Then, we compared the observed abundance profiles of neighboring spots with the expected abundance profiles, which were calculated by multiplying the number of neighboring spots by the average abundance profiles across all spots in the spatial transcriptomic data. The observed-to-expected abundance profile ratio was defined as the enrichment profile. The cell–cell pairs with mutual enrichment (observed-to-expected ratio > 1) alone were counted in the neighborhood graph.

Additional materials and methods can be found in Additional file 3: Supplementary materials and methods.

## Results

### Generating a comprehensive single-cell transcriptomic landscape of pancreatic cancer cells and CAFs

**Fig. 1:**
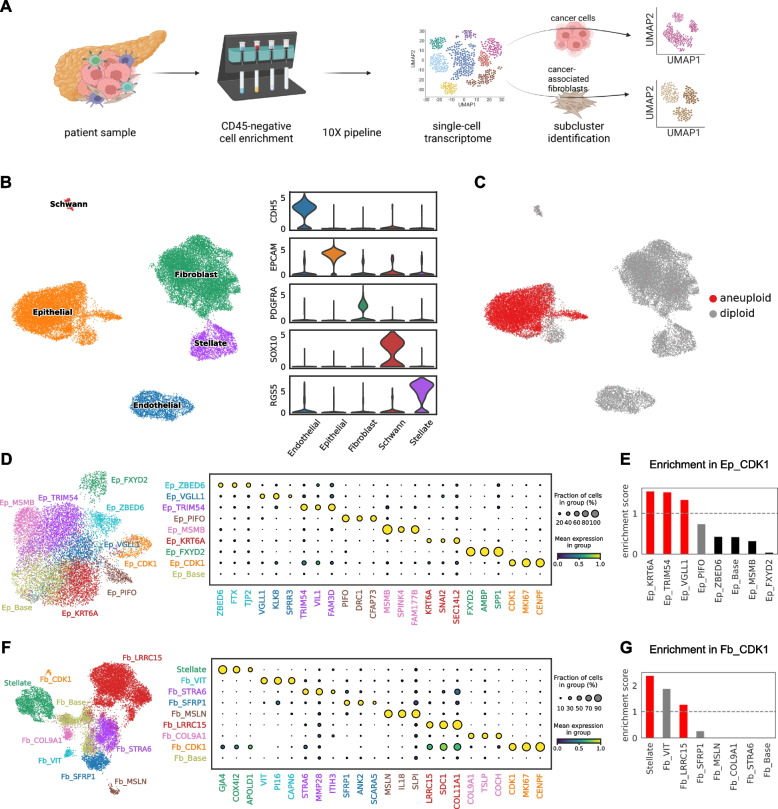
Single-cell transcriptomic landscape of epithelial cells and fibroblasts in pancreatic cancer.** A** Experimental workflow and data preprocessing steps. **B** UMAP projection of five major cell populations identified in the CD45-negative cell population of pancreatic cancer. **C** Predicted copy number alterations across the major cell populations. **D** UMAP projection of epithelial cell subclusters with their specific markers. **E** Relative abundance of epithelial subclusters in the proliferating population. Enrichment scores were calculated by dividing each subcluster’s fraction in the proliferating epithelial population (Ep_CDK1) by the fraction in the non-proliferating population. Bar colors indicate the significances tested by proportion *z*-tests: red (significant enrichment), black (significant depletion). **F** Fibroblast-stellate cell subclusters with their specific markers. **G** Relative abundances of fibroblast-stellate subclusters in the proliferating population as in **E**

To account for the heterogeneity of cancer cells and CAFs, we conducted single-cell RNA sequencing with CD45-negative enriched cells from surgical samples of 17 pancreatic tumor patients (16 pancreatic cancer and one HGD with IPMN pathology) (Fig. 1A). We identified five major cell types: epithelial cells, fibroblasts, Schwann cells, endothelial cells, and stellate cells, from the whole dataset (Fig. 1B and Additional file 1: Table S2). We then identified cancer cells using a copy number alteration inference tool [27], and we found that the predicted malignant cells were located in the epithelial cell population (Fig. 1C), as expected. Next, we sub-clustered the epithelial cell and fibroblast population for further characterization.

### Characterization of the mutational and cycling profiles of pancreatic cancer cell clusters

Pancreatic cancer epithelial cells were initially separated into nine clusters, which could be identified by specific marker genes (Fig. 1D, Additional file 1: Table S3, and Additional file 2: Fig. S2A-C). Among the marker genes, we selected the most representative marker gene for each cluster based on the expression specificity. Each epithelial cluster was named by combining the representative marker gene name with the “Ep_” prefix, except for the Ep_Base cluster, for which the marker gene was not identified and excluded in downstream analysis. The expression specificities of the subcluster markers were conserved in each sample (Additional file 2: Fig. S2D). We also integrated the publicly available pancreatic cancer scRNA-seq dataset [13] with ours and validated the existence of each population (Additional file 2: Fig. S2E).

To check the mutational status of the epithelial subclusters, we evaluated the sequences of the G12 site of KRAS gene, which is the most common driver mutation site for pancreatic cancer (Additional file 2: Fig. S3A-C). Unlike other epithelial clusters, most of the cells in Ep_FXYD2 cluster had wild-type KRAS sequences. In addition, Ep_FXYD2 cells were predicted to contain diploid genomes in the copy number inference analysis (Additional file 2: Fig. S3D-G). As the KRAS mutation is regarded as one of the earliest events during PDAC progression [28], we assumed Ep_FXYD2 cells represent non-cancerous epithelial cells. Recently discovered markers of premalignant ductal cells, including FXYD2 [29], suggest this population’s identity as a premalignant ductal-like cell (Additional file 2: Fig. S3H).

The Ep_CDK1 cluster represents proliferating cancer epithelial cells, which can be identified with prominent cell cycle signatures (Fig. 1D and Additional file 2: Fig. S4A). The absence of cycling profiles in all other epithelial subclusters indicates a convergence of cycling cells from different epithelial subclusters, due to the strong transcriptomic changes accompanied in the cell proliferation process. Thus, we tried to identify the original identities of cells composing Ep_CDK1 cluster by training a logistic regression model from epithelial cells other than the Ep_CDK1 cluster (Additional file 2: Fig. S4B). The validity of the transferred annotation was confirmed by differential expression of the marker genes within the proliferating cells (Additional file 2: Fig. S4C). Next, we compared the ratio of cell proportions in the cycling cluster to total epithelial cells (Additional file 2: Fig. S4D-E). Among cancer cell clusters, Ep_TRIM54, Ep_KRT6A, and Ep_VGLL1 were enriched in the cycling cell population (Fig. 1E), which implies that these epithelial subclusters are relatively more proliferative than the others. In summary, our analysis identified six different cancer cell subclusters, excluding premalignant Ep_FXYD2 and cycling Ep_CDK1 clusters.

### Characterizing pancreatic cancer-associated fibroblast populations

Clustering pancreatic cancer fibroblasts initially separated them into eight different populations (Fig. 1F). Similar to cancer cells, we annotated fibroblast clusters with a systematic naming system by combining the specific marker gene with a “Fb” prefix (Additional file 1: Table S3 and Additional file 2: Fig. S5A-C). Except for the Fb_Base cluster, we found specific marker gene sets for each fibroblast cluster (Fig. 1F and Additional file 2: Fig. S5D). Excluding the Fb_Base and cycling Fb_CDK1 populations, our analysis resulted in six distinct pancreatic CAF clusters with unique gene expression profiles. We checked the average expression of CAF signatures, suggested by previous studies [11, 30], in our fibroblast clusters (Additional file 2: Fig. S6A-B). This analysis showed a near-exact match between Fb_SFRP1: iCAF (inflammatory CAF), Fb_LRRC15: myCAF (myoblastic CAF), and Fb_MSLN: apCAF (antigen-presenting CAF).

To further characterize the less-characterized fibroblast populations, we first utilized the signatures suggested from the recent pan-tissue fibroblast atlas dataset [31]. Interestingly, marker genes of Fb_VIT were highly expressed in the Pi16 + fibroblasts, which represent the global fibroblast progenitor population (Additional file 2: Fig. S6C-F). Reciprocally, we evaluated the expression of PI16 and CD34, the marker genes of Pi16 + fibroblasts, in our fibroblast dataset and found that they are highly expressed in Fb_VIT cells (Additional file 2: Fig. S6G). Notably, the progenitor markers were also expressed in the Fb_SFRP1 population at a moderate level, and streamlines from Fb_VIT to Fb_SFRP1 were shown in RNA velocity map (Additional file 2: Fig. S6G-H). These results suggest the existence of a global fibroblast population in the PDAC tumor microenvironment (TME) and their potential contribution to the iCAF population. Using RNA in situ hybridization images, we validated the existence of Fb_VIT in human PDAC tissues (Additional file 2: Fig. S7). We also investigated core features of PDAC sub-TMEs from a recent study [32], which identified “reactive” sub-TME, containing plump fibroblasts with enlarged nuclei, and “deserted” sub-TME, featuring loose mature fibers, in human PDAC tissues. The core features of deserted sub-TMEs were highly enriched in the Fb_STRA6 cluster (Additional file 2: Fig. S6I).

Similar to the Ep_CDK1 cluster in cancer cells, we analyzed the Fb_CDK1 cluster by transferring annotation from non-cycling fibroblast clusters (Additional file 2: Fig. S8A-C). The analysis marked stellate cells and Fb_LRRC15 as major proliferating clusters in pancreatic fibroblast populations (Fig. 1G and Additional file 2: Fig. S8D-E). To summarize, we identified six distinct CAF clusters, including three well-described CAF populations: Fb_SFRP-iCAF, Fb_LRRC15-myCAF, and Fb_MSLN-apCAF, and three less-described CAF populations: Fb_VIT-global fibroblasts, Fb_STRA6-fibroblasts in deserted sub-TME and Fb_COL9A1.

### Population-based clustering identifies pathological and molecular subtypes of pancreatic cancer

**Fig. 2:**
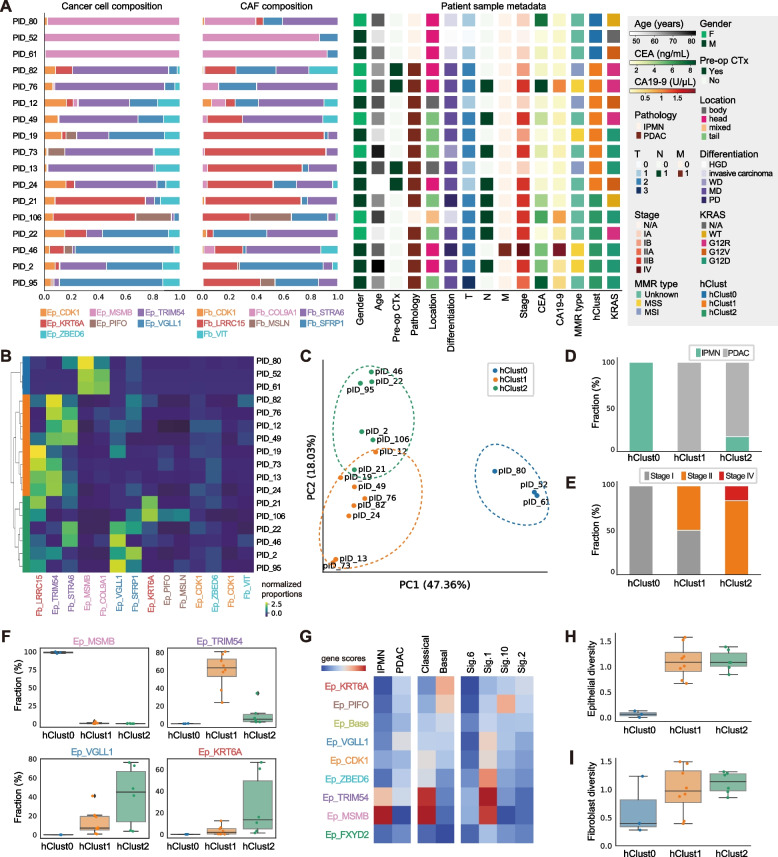
Population-based clustering identifies pathological and molecular subtypes of pancreatic cancer. **A** Bar plots and heatmaps displaying the subcluster composition and clinical information of the patients included in this study. **B** Heatmap representation of the hierarchical clusters of pancreatic cancer patients. **C** PCA plot of the patient hierarchical clusters. The hierarchical clustering and PCA were based on the composition data for the cancer cell population and CAF population. **D** Bar plot representing the fraction of samples pathologically diagnosed as IPMN or PDAC across the patient clusters. **E** Pathological stages of the patients in each patient subcluster. **F** Proportions of cancer cell clusters showing differential patterns in their fraction across the patient clusters. **G** Heatmap showing the average expression of signature genes in pancreatic cancer subtypes (IPMN—adenocarcinoma, classical—basal-like, NMF signatures). **H,I** Shannon Diversity Index was calculated in (**H**) the cancer cell population and (**I**) the CAF. Whiskers indicate minimum and maximum values, and values exceeding 1.5 × IQR (interquartile range) are noted as outliers

Having identified detailed subclusters of cancer cell and CAF populations in pancreatic cancer tissue, we next questioned whether we could stratify patients based on the composition of these single-cell-based clusters (Fig. 2A and Additional file 1: Table S4). We performed hierarchical clustering on the proportions of cancer cell and CAF clusters, which resulted in three separate patient groups (hClust0-2) (Fig. 2B,C). By comparing clinical metadata, we found that three out of four IPMN samples were included in hClust0, and advanced disease status were increasingly enriched in hClust1 and hClust2 (Fig. 2D,E). We noted that each patient cluster can be marked by prominent enrichment of specific cancer cell clusters: Ep_MSMB for hClust0, Ep_TRIM54 for hClust1, and Ep_KRT6A and Ep_VGLL1 for hClust2 (Fig. 2F and Additional file 2: Fig. S9A-C). To validate the robustness of this patient clustering result, we merged 57 tumor scRNA data from 4 different cohorts including our study [13, 14, 33]. We re-annotated cancer cells and CAFs from the merged dataset and performed unsupervised clustering, which confirmed the same clustering pattern in a larger cohort (Additional file 2: Fig. S9D-H).

To further characterize the patient clusters, we compared the expression of gene signatures that are highly associated with pancreatic cancer subtypes (Fig. 2G). First, we found high levels of IPMN-associated genes [34] in Ep_MSMB cancer cells. This is consistent with the enrichment of IPMN pathology samples in hClust0. We also noted that the population diversity of hClust0 was much lower than in other patient clusters (Fig. 2H,I), and the CAF populations in hClust0 patients were dominated by Fb_COL9A1, which was further validated using in situ hybridization images and immunohistochemistry (Additional file 2: Fig. S10A-B). These results indicate that hClust0 represents the IPMN pathology in pancreatic cancer, and IPMNs can be distinguished by unique cancer epithelial cell and CAF composition: Ep_MSMB and Fb_COL9A1.

Next, we utilized the gene expression signatures suggested for the classification of classical and basal-like pancreatic cancer [7]. We found that Ep_TRIM54 and Ep_KRT6A express high levels of classical and basal-like gene signatures, respectively (Fig. 2G). In accordance with a worse prognosis in basal-like pancreatic cancer patients [35], the Ep_KRT6A-high hClust2 had more patients with advanced cancer stages (Fig. 2E). In addition to Ep_KRT6A, Ep_PIFO was also highly associated with basal-like cancer signatures. The existence of the Ep_PIFO population in pancreatic cancer tissue is highly supported by a bulk NMF signature (Signature 10) from a recent study [15] (Additional file 2: Fig. S9I-J), and the marker genes indicate that this cluster is highly correlated to cilia functions [36–38]. No significant correlations between cancer cell subclusters and CAF subclusters were found in this study. Nonetheless, using the signatures from the previous pancreatic cancer studies, we identified cancer cell subcluster representing classical (Ep_TRIM54) and basal-like (Ep_KRT6A and Ep_PIFO) subtypes of pancreatic cancer, along with the cancer subcluster highly correlated to IPMN pathology (Ep_MSMB).

### Ep_VGLL1 is a new pancreatic cancer cell population associated with poor prognosis

Next, we evaluated the prognostic value of cancer and CAF clusters using public bulk transcriptome data from TCGA and ICGC. Since bulk transcriptomes contain gene expression profiles from various cell types, we refined our marker gene set to ensure that the refined marker gene set does not include genes that are expressed in other cell types (Additional file 1: Table S5 and Additional file 2: Fig. S11A-B). To analyze the immune cell expression profile in pancreatic cancer, we additionally generated scRNA-seq data from three patients included in this study through CD45-positive enrichment (Additional file 2: Fig. S12A-B). Samples in each cohort were divided into two groups according to the marker gene expression with optimal cut-off values (Additional file 2: Fig. S11C) [25]. We then evaluated whether the two groups are showing different survival patterns.

**Fig. 3:**
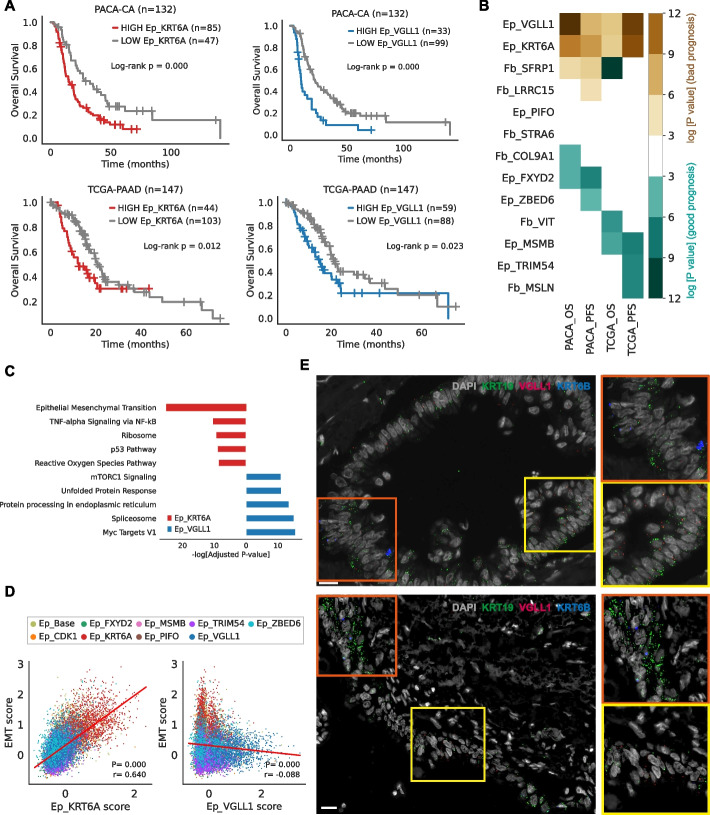
Identification of subpopulations with prognostic values.** A** Kaplan–Meier survival curves representing the overall survival of patients included in ICGC (PACA-CA) and TCGA (PAAD), stratified by the expression level of the Ep_KRT6A signature and Ep_VGLL1 signature. *P*-values were determined by log-rank tests. **B** Prognostic values of cluster-specific markers in two public cohorts. Colors indicate log-transformed *P*-values, and *P*-values were determined by log-rank tests comparing high- and low- expression groups. Dark green color indicates favorable prognosis in the high-expression group, whereas brown color indicates worse prognosis in the high-expression group compared to the low-expression group. **C** Results from the pathway enrichment analysis conducted on the DEGs comparing Ep_VGLL1 and Ep_KRT6A. For the DEG analysis, the Wilcoxon rank-sum test was used for statistical testing with adjusted *P-*value cut-off 0.05. **D** Scatter plots showing the correlation between EMT scores and Ep_KRT6A or Ep_VGLL1 scores across pancreatic cancer cells. EMT scores were calculated using the EMT signature gene set and subcluster scores calculated by the expression of subcluster-specific genes. **E** RNA in situ hybridization images from human pancreatic cancer tissue. Green, red, and blue colors indicate KRT19, VGLL1, and KRT6B, respectively. Orange and yellow boxes highlight KRT6B and VGLL1 expressing tumor epithelial cells, respectively

Among all the epithelial and fibroblast subpopulations, only the markers of Ep_KRT6A and Ep_VGLL1 consistently marked prognostic significance, and they denoted bad prognosis (Fig. 3A,B and Additional file 2: Fig. S11D). The bad prognosis of the high Ep_KRT6A group was predictable, due to its basal-like character, unlike Ep_VGLL1 cluster, which did not show any specific pattern of previously defined signatures (Fig. 2G). We confirmed that the Ep_VGLL1 score consistently demonstrated prognostic significance in both univariate and multivariate analyses (Additional file 1: Table S6). The pathway enrichment analysis clearly showed that Ep_KRT6A expressed higher levels of epithelial-to-mesenchymal transition (EMT) gene sets, compared to Ep_VGLL1 (Fig. 3C and Additional file 2: Fig. S11E). Also, Ep_KRT6A score showed a clear positive correlation with the EMT score across the cancer cells, whereas Ep_VGLL1 score did not correlate at all (Fig. 3D).

In addition, the differential marker gene expression pattern across the public cohorts distinguished Ep_VGLL1 from Ep_KRT6A cells. Though the Ep_KRT6A and Ep_VGLL1 scores correlated within classical pancreatic cancer samples, their correlations were very weak in basal-like samples (Additional file 2: Fig. S11F-K). Also, we found that only the proportion of Ep_VGLL1 correlated with the serum CA 19–9 level (Additional file 2: Fig. S11L). Most importantly, RNA in situ hybridization images from human PDAC tissue clearly separated VGLL1-expressing cells and KRT6B-expressing cells (Fig. 3E). The cancer cell cluster markers were also validated in immunohistochemistry images (Additional file 2: Fig. S13A-D).

Thus, our analysis identified two biologically distinct cancer cell types, Ep_VGLL1 and Ep_KRT6A, that are associated with bad prognosis.

### Deciphering the transcription factor network regulating pancreatic cancer cell clusters

**Fig. 4:**
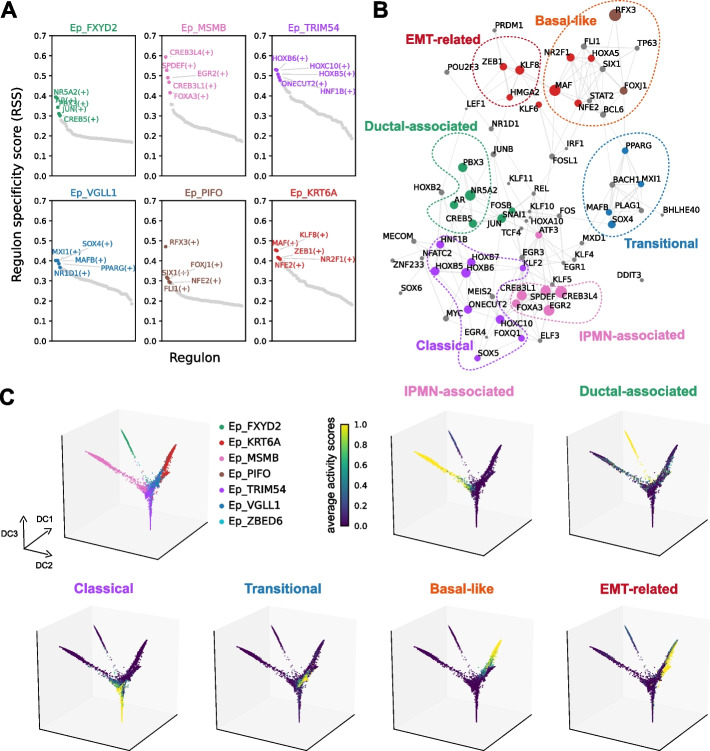
Transcription factor network regulating pancreatic cancer cell clusters.** A** Specific transcription factor activities across cancer cell clusters. The top five transcription factors showing specific activities for each cancer cell cluster are shown. **B** Transcription factor network in the pancreatic epithelial cell population. Edge widths are proportional to the correlation coefficients between the transcription factor pairs. Node colors indicate the cancer cell clusters associated with, and the size is proportional to the significance of the association. **C** 3D Diffusion maps based on the transcription factor activities. The activities of transcription factors in each TF cluster were averaged into a single score and projected onto the 3D diffusion map

To identify the biological characteristics of cancer cell clusters, we calculated the transcription factor (TF) activities in each cancer cell cluster using SCENIC [39, 40] pipeline (Fig. 4A). We then reconstructed a correlation-based TF network based on the TF activity data (Fig. 4B). Notably, the inferred activities of TFs were grouped into distinct clusters that correlated well with the epithelial clusters except for Ep_ZBED6 (Fig. 4A, Additional file 1: Table S7, and Additional file 2: Fig. S14A), which indicates the unstable nature of the subcluster. We named each group of TFs based on the characteristics of the corresponding epithelial clusters (Fig. 4B and Additional file 2: Fig. S14B-C).

As we identified Ep_FXYD2 as a premalignant ductal-like population, we annotated a group of TFs corresponding to Ep_FXYD2 as “Ductal-associated.” As expected, NR5A2, an essential element for constraining pancreatic cancer initiation [41], was included in the “Ductal-associated” TF cluster. Similarly, the TF cluster that exhibited high activity in Ep_MSMB was dubbed as “IPMN-associated.” SPDEF, a key regulator of mucin production and a tumor suppressor for colorectal and prostate cancer [42, 43], was assigned to this “IPMN-associated” TF cluster. The activities of the “classical” TFs were high in Ep_TRIM54, in contrast to the “basal-like” TFs that were highly expressed in the Ep_KRT6A and Ep_PIFO cells (Additional file 2: Fig. S14C). Notably, the “classical” TF cluster included HNF1B and ONECUT2 (HNF-6β), which has been suggested to be one of the key elements in the classical subtype of PDAC [15]. TP63 and SIX1 in the “basal-like” TF cluster were also shown to be an essential element in the basal-like program of PDAC [44–46]. Connected to the “basal-like” TF cluster was the “EMT-related” TF cluster, which contains key regulators of the EMT process, such as ZEB1. The “EMT-related” TF cluster was highly specific to the Ep_KRT6A population, highlighting its strong association with the EMT process.

Finally, we identified a set of TFs that are highly specific to the Ep_VGLL1 cluster (Fig. 4A). These TFs were positioned in the junction connecting “IPMN-associated” / “Ductal-associated” / “classical” groups with the “basal-like” groups (Fig. 4B). This pattern was also reproduced in the diffusion map and PCA projection based on TF activity, which positions this TF cluster at the midpoint of “classical” and “basal-like” clusters (Fig. 4C and Additional file 2: Fig. S14D). Based on these characteristics, we postulated that Ep_VGLL1 represents a transitional cancer cell population connecting classical and basal-like cancer cells, and annotated the Ep_VGLL1-specific TFs as “transitional” TFs.

### Molecular features of Ep_VGLL1 suggest its transitional property

**Fig. 5:**
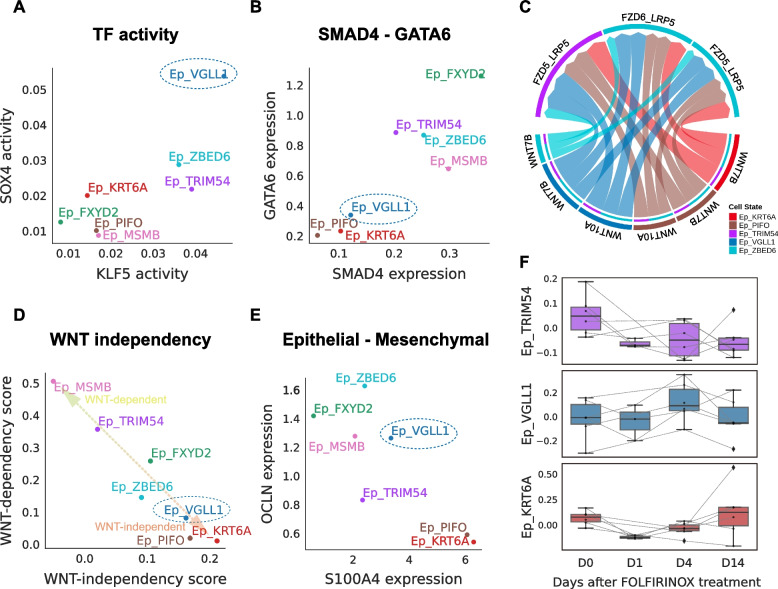
Ep_VGLL1 represents the transitional cancer cell population in PDAC progression. **A** Average inferred transcription factor activities of KLF5 and SOX4 across the epithelial cell clusters in pancreatic cancer. **B** Average expression of SMAD4 and GATA6 in epithelial cell clusters. **C** Wnt signaling network in the epithelial cell population. **D,E** Scatter plots showing the average (**D**) Wnt dependency and Wnt independency scores and (**E**) S100A4 and OCLN expression across the epithelial cell clusters. **F** Expression of epithelial subcluster markers in FOLFIRINOX-treated pancreatic cancer tumor spheroid cells. The tumor spheroid cells were derived from six different patients and the expression data downloaded from a previous study [9]. Whiskers indicate minimum and maximum values, and values exceeding 1.5 × IQR (interquartile range) are noted as outliers

From the TF analysis results, we noted that Ep_VGLL1 showed a distinct level of SOX4 and KLF5 activities (Fig. 5A). Since the cooperation between SOX4 and KLF5 has been suggested as a molecular mechanism that drives tumorigenesis in SMAD4 defect pancreatic cancer cells [47], we postulated that Ep_VGLL1 represents cancer cells in SMAD4-deficient oncogenic processes driven by TGF-β. Accordingly, we found that the average expression of SMAD4 was low in Ep_VGLL1, in a level comparable to the basal-like clusters (Fig. 5B), and these features suggest a basal-like property [15] of Ep_VGLL1. Interestingly, expression of TGF-β-induced apoptosis genes [48] were high in Ep_ZBED6 (Additional file 2: Fig. S15A), indicating that Ep_ZBED6 cells represent cancer cells undergoing TGF-β-induced apoptosis. Meanwhile, low expression of GATA6 (Fig. 5B), a well-known surrogate marker of classical PDAC [10, 49], in Ep_VGLL1 also suggests the non-classical nature of Ep_VGLL1. As GATA6 expression is known to correlate with Wnt signaling dependency in pancreatic cancer cells [50], reduced GATA6 expression suggests a Wnt-independent nature of Ep_VGLL1. Accordingly, cell-to-cell signaling analysis [51] reveals that Ep_VGLL1 could participate in the Wnt signaling pathway as a sender (Fig. 5C). Using the previously reported gene signatures of Wnt dependency [50], we found that Ep_VGLL1 had strong Wnt-independent signatures like other basal-like clusters (Fig. 5D).

We also investigated the cellular identity of Ep_VGLL1 because KLF5 is an essential element for maintaining epithelial cell characteristics in various cell types [52–56]. In accordance with its high KLF5 activity, Ep_VGLL1 highly expressed the tight junction protein genes TJP1 and OCLN. Also, Ep_VGLL1 expressed low levels of mesenchymal cell markers VIM and S100A4, unlike other basal-like clusters (Fig. 5E and Additional file 2: S15B-C). These results describe the intermediate and intriguing nature of Ep_VGLL1 cluster, acquiring TGF-β resistance and Wnt-independence but still maintaining epithelial cell characteristics.

Recently, classical to basal-like transition was reported to occur in pancreatic cancer tumor spheroid cells under FOLFIRINOX treatment through cancer-intrinsic mechanisms [9]. We traced this transition process with our own subcluster markers. As expected, the Ep_TRIM54 signature showed a decreasing pattern along the time axis, whereas the Ep_KRT6A signature increased over time (Fig. 5F and Additional file 2: Fig. S15D). Surprisingly, the Ep_VGLL1 signature peaked at day 4 (D4), right before the Ep_KRT6A surge, and then decreased gradually. These results suggest that this subcluster with intermediate features, Ep_VGLL1, might represent a transitional population between basal-like and classical types of cancer cells.

### Spatial transcriptomic data recapitulate dynamics of cancer subclusters and cancer-associated niches in human pancreatic cancer

**Fig. 6:**
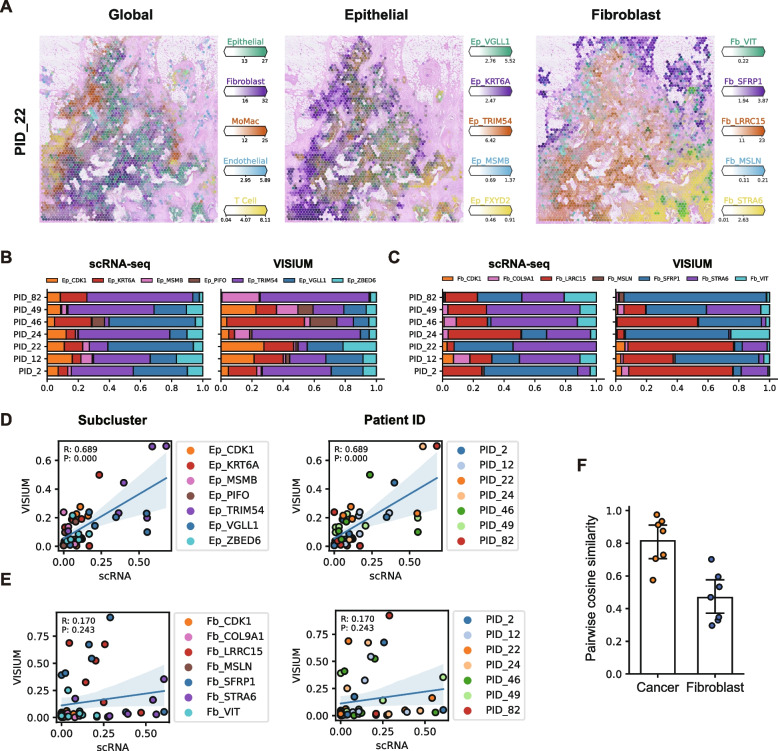
Spatial deconvolution of human PDAC tissue.** A** Predicted cellular abundances in spatial transcriptome data from a PDAC patient sample (PID_22). Major global cell types, major epithelial, and fibroblast subclusters are shown. **B,C** Subcluster compositions of (**B**) cancer cell and (**C**) fibroblast populations in PDAC patient samples. **D,E** Scatter plots depicting subcluster compositions of (**D**) cancer cell and (**E**) fibroblast populations from scRNA-seq and paired spatial data. Each dot represents the proportions of each subcluster in a patient, where the proportion from scRNA-seq data is plotted on the *x*-axis, and the proportion from the paired spatial data is plotted on the *y*-axis. Pearson’s *r*-value and *P*-value for the correlation coefficient are depicted on the upper left side of each plot. **F** Pairwise cosine similarities of cancer cell and fibroblast subcluster compositions

Using the detailed reference map of cancer cell and CAF clusters, we attempted to resolve the spatial complexities of human pancreatic cancer. Accordingly, we generated paired spatial transcriptomic data (*n* = 7) from the scRNA-seq cohort. Using our scRNA-seq data as a reference (Additional file 2: Fig. S16A–B), we deconvoluted the spatial transcriptomic data using a Bayesian inference model [26], which identified the spatial localization pattern of global cell types, cancer cell subclusters, and fibroblast subclusters (Fig. 6A and Additional file 2: Fig. S17-18). Subsequently, we compared subcluster compositions between the scRNA data and paired spatial transcriptomic data for cancer cell (Fig. 6B) and fibroblast subclusters (Fig. 6C). We found that cancer subcluster compositions from the spatial transcriptomic data highly corresponded to the cancer cell compositions from the paired scRNA data (Fig. 6D), unlike fibroblast compositions (Fig. 6E,F). Since spatial transcriptomic data contain information of a tissue section, these results imply a minimum level of intra-tumor spatial heterogeneities in cancer cell compositions.

**Fig. 7:**
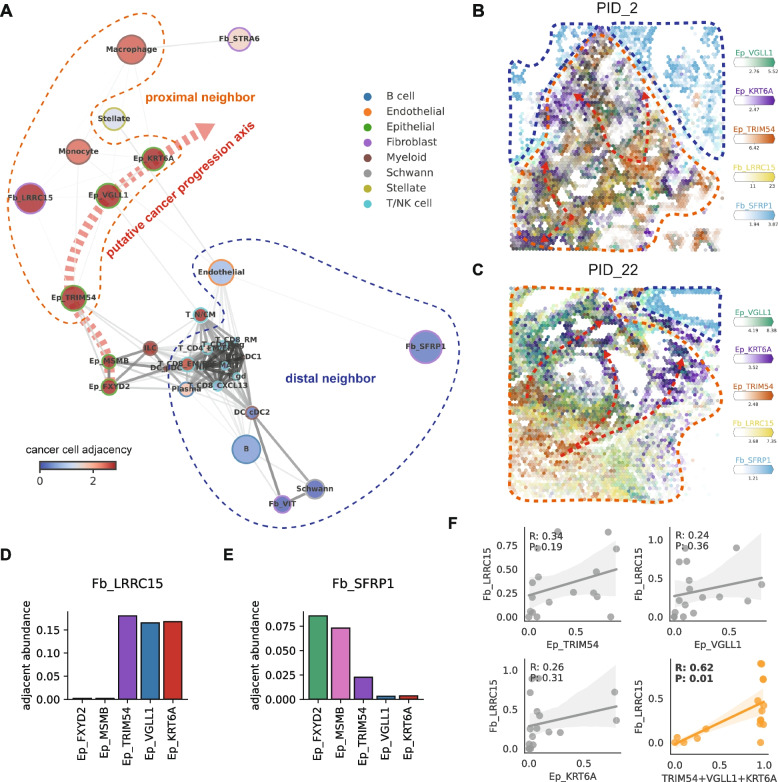
Identification of niches in human PDAC tissue.** A** Neighborhood graph representing neighborhood enrichment of cell types. Edges represent average neighborhood enrichment scores (observed-to-expected ratio) between the cell types, and only the bidirectional enrichments were depicted in this graph as edges. Dot sizes are proportional to the estimated abundances (log scale), and the colors represent average cancer cell abundances in each cell type’s neighborhood. **B**,**C** Representative images of deconvoluted spatial transcriptome data from two PDAC patients, colored with the abundances of three major cancer cell subclusters and two major fibroblast subclusters of PDAC. Orange dashed lines indicate cancer proximal niches, while blue dashed lines indicate cancer distal niches and the red dashed lines indicate putative cancer progression axis. **D**,**E** Average estimated abundances of the two major fibroblast subclusters, (**D**) Fb_LRRC15 and (**E**) Fb_SFRP1, in each epithelial subcluster’s neighborhood. **F** Correlation between the fraction of Fb_LRRC15 in the fibroblast population and the fraction of major cancer cell clusters in scRNA-seq data. Pearson’s *r*-value and *p*-value are denoted on the upper left corner of each plot

To decipher the relationships between the spatial distribution patterns of these diverse subtypes of cancer cells, fibroblasts, and other major cellular components in human pancreatic cancer, we calculated the observed-to-expected ratio of neighborhood cell compositions and constructed an adjacency network graph reflecting the neighborhood enrichment relationships across diverse cell types (Fig. 7A). Notably, we found that Ep_VGLL1 was spatially correlated with both Ep_TRIM54 and Ep_KRT6A whereas these two major populations representing classical and basal-like subtypes of pancreatic cancer, respectively, were not directly adjacent to each other. This spatial relationship strongly supports our model of pancreatic cancer dynamics, which identifies Ep_VGLL1 as a bridging population between classical and basal-like subtypes of pancreatic cancer.

Furthermore, we focused on the distributions of stromal and immune cells and their spatial correlations with cancer cell clusters. The adjacency network graph distinguished cell types that were located at the proximities of cancer cells from those that were not (Fig. 7A–C and Additional file 2: Fig. S17). We discovered that the spatial distributions of Fb_LRRC15, which represents the myCAF population, were highly correlated with pancreatic cancer cell distributions (Fig. 7D), whereas Fb_SFRP1 cells, representing iCAF, were located distal to the cancer spots (Fig. 7E), as can be expected from previous studies [57, 58]. Intriguingly, we found that none of the major cancer cell subclusters (Ep_TRIM54, Ep_VGLL1, and Ep_KRT6A) exhibited preferences for Fb_LRRC15 (Fig. 7D). This is consistent with the results from the scRNA-seq data, where none of the Ep_TRIM54, Ep_VGLL1, or Ep_KRT6A showed correlation with Fb_LRRC15 individually, whereas the sum of these populations was highly correlated with the proportion of Fb_LRRC15 (Fig. 7F). Overall, the investigation of human PDAC spatial transcriptomic data revealed spatial associations between the cancer cell subclusters, which supports the suggested transitional properties of Ep_VGLL1, and also revealed cellular components of tumor-proximal and tumor-distal niches in human PDAC tissues.

## Discussion

A comprehensive landscape of pancreatic cancer cells presented in this study encompasses many facets of pancreatic cancer diversity. The major molecular subtypes of pancreatic cancer, classical and basal-like (or quasi-mesenchymal), are represented in this single-cell transcriptome landscape as Ep_TRIM54 and Ep_KRT6A, respectively. We showed here that Ep_KRT6A is highly correlated with EMT features, in accordance with a previous report [15]. We also newly identified a basal-like subcluster with unique cilia-related features, Ep_PIFO, whose features were suggested in a couple of recent studies [15, 38], without clear demarcation in single-cell datasets. Cancer cells highly associated with IPMN pathology were also clearly identified in this study as a distinct cluster, Ep_MSMB. Our landscape of epithelial cells also includes the premalignant ductal-like population [29] which we dubbed Ep_FXYD2. This spectrum of pancreatic cancer cells in our epithelial landscape could provide a robust framework when analyzing pancreatic cancer cell diversities.

We also discovered a novel pancreatic cancer cell population whose character could not be assigned to either basal-like or classical, despite the prognostic value of its marker genes. The population, Ep_VGLL1, shares some cardinal cellular features with classical cells, expressing high levels of tight junction genes (TJP1 and OCLN) and low levels of mesenchymal markers (VIM and S100A4). On the other hand, low expression of SMAD4 and GATA6 suggest that Ep_VGLL1 shares dysregulatory features with basal-like clusters (i.e., Ep_KRT6A and Ep_PIFO) rather than the classical cluster. Interestingly, TF activity analysis showed that Ep_VGLL1 can be distinguished by the high activities of KLF5 and SOX4, the TF pair marking the tumorigenic SMAD4-deficient pancreatic cancer cells under the TGF-β milieu [47].

Together with these intriguing features of Ep_VGLL1, some indirect evidence, including the inferred TF network (Fig. 4B,C) and the differential marker gene expression pattern in a dataset featuring classical to basal-like transitions (Fig. 5F), suggest putative classical to basal-like transitions through Ep_VGLL1. Most importantly, we discovered that Ep_VGLL1 was spatially correlated with both classical (Ep_TRIM54) and basal-like cancer (Ep_KRT6A) clusters (Fig. 7A). Recent studies reporting the intratumoral co-existence of basal-like and classical type cancer cells [12, 15] and some in vitro experiments [9, 59] support the possibilities of the classical to basal-like transition. Direct evidence of classical to basal-like transitions is still lacking, and it would be addressed in future studies.

The dismal prognosis of pancreatic cancer is attributed to its aggressive biological behavior and the acquisition of early resistance to chemotherapy. Approximately 31 and 23% of patients responded to the current primary regimen, FOLFIRINOX and gemcitabine + nab-paclitaxel, respectively. However, even among these responders, drug resistance typically develops within 6 months, leading to disease progression [3, 5]. The mechanism of acquiring drug resistance remains unclear; however, emerging evidence suggests that early acquisition of drug resistance to chemotherapy in pancreatic cancer cells is associated with the acquisition of an EMT-like phenotype [60–65]. Several studies have demonstrated the therapeutic potential of inhibiting EMT to overcome chemoresistance [61, 66, 67]. Herein, we identified a novel cell cluster, Ep_VGLL1, which appears to emerge during the transition from the classical to basal-like subtypes. Since basal-like properties are highly correlated with EMT programs [15] (Fig. 3D), targeting Ep_VGLL1 to interrupt the classical to basal-like subtype transition would be a promising novel therapeutic strategy for overcoming chemoresistance in pancreatic cancer. To better link the Ep_VGLL1 population with the therapeutic responses, cost-effective detection methods based on the markers discovered in this study should be developed to allow the future large cohort studies.

Cancer-associated fibroblast (CAF) is another highly variable population in pancreatic cancer. Major CAF subpopulations were recently identified in the mouse model of PDAC [11, 30]. Although they validated the existence of CAF subpopulations in human PDAC samples, minor subpopulations other than myCAF and iCAF (or TGF-β-CAF and IL1-CAF, respectively) were hardly identifiable as distinct subpopulations in human PDAC samples. In this study, through the cell enrichment process, we successfully identified minor CAF populations, along with previously defined major CAF subpopulations: myCAF-Fb_LRRC15, iCAF-Fb_SFRP1, and apCAF-Fb_MSLN. Newly identified minor CAF subclusters included a global fibroblast progenitor population [31], which we annotated as Fb_VIT. This population may represent another source of the PDAC CAF population beside stellate cells [57] and mesothelial cells [68]. This finding is in line with a recent study reporting the limited contribution of stellate cells to the CAF population [69]. We also identified fibroblast subclusters highly associated with IPMN pathology (Fb_COL9A1) and deserted sub-TME in PDAC pathology (Fb_STRA6).

By analyzing spatial transcriptomic data, we identified tumor-associated niches in human PDAC tissue. Tumor-proximal niches were enriched with tumor-infiltrating myeloid cells and myCAF (Fb_LRRC15) and tumor-distal niches were occupied by majority of the immune cells and iCAF (Fb_SFRP1). These results not only confirm previous reports [57, 58], but also provide detailed insights such as the universal association of myCAF with cancer subtypes. The mechanism of interaction between the newly identified cell types and their impact on PDAC tissue microenvironments awaits further investigation.

## Conclusions

In conclusion, this study provides a comprehensive analysis of epithelial cells and fibroblasts in the pancreatic cancer tumor microenvironment through a deep single-cell transcriptome analysis. We identified a new epithelial cell cluster with prognostic value and developed a novel framework for the pancreatic cancer cell dynamics. Regarding the clinical implication of the molecular subtype of pancreatic cancer, this detailed dissection of cancer cells and stromal cells in pancreatic cancer provides the basis for developing a novel therapeutic strategy to overcome chemoresistance and ultimately improving the prognosis of pancreatic cancer patients.

### Supplementary Information

**Additional file 1: Table S1. **Clinical characteristics of the patients (*N*=17). **Table S2.** Marker genes for major cell types. **Table S3.** Marker genes for subclusters. **Table S4.** Cell type proportion data. **Table S5.** Refined marker gene set based on the expression specificity against all other clusters. **Table S6.** Univariate and multivariate Cox regression analysis of overall survival in the pancreatic cancer patients from the TCGA cohort. **Table S7.** Transcription factor activities according to each epithelial cluster.

**Additional file 2: Fig. S1. **Representative histology of patient samples. **Fig. S2.** Identification of the epithelial subpopulations in pancreatic cancer. **Fig. S3.** Identification of the malignant populations in pancreatic cancer epithelial cells. **Fig. S4.** Deconvolution of the proliferating epithelial subpopulation. **Fig. S5.** Identification of the fibroblast subpopulations in pancreatic cancer. **Fig. S6.** Integration of the fibroblast atlas identifies a fibroblast progenitor population in pancreatic cancer. **Fig. S7.** Identification of Fb_VIT populations. **Fig. S8.** Deconvolution of the proliferating fibroblast subpopulation. **Fig. S9.** The composition of cancer cell and CAF subpopulations across patient clusters. **Fig. S10.** Identification of Fb_COL9A1 populations. **Fig. S11.** A strategy to identify marker gene sets with prognostic values in PDAC. **Fig. S12.** Immune cells in human pancreatic cancer tissue. **Fig. S13**. Immunohistochemistry (IHC) images of the major cancer cell markers. **Fig. S14.** Correlation between the TF clusters and epithelial subclusters. **Fig. S15.** Cellular characteristics of the Ep_VGLL1 population. **Fig. S16.** Reference single-cell transcriptome dataset for spatial deconvolution. **Fig. S17.** Representative images of spatial deconvolution of human pancreatic cancer. **Fig. S18.** Marker gene expressions in spatial transcriptome data.

**Additional file 3. **Supplementary materials and methods.

## Data Availability

RNA sequencing data is uploaded on Gene Expression Omnibus (GEO) database. Raw sequence file of the non-immune PDAC scRNA-seq data is available from NCBI with accession number GSE194247 (https://www.ncbi.nlm.nih.gov/geo/query/acc.cgi?acc=GSE194247) [70], and GSE235449 (https://www.ncbi.nlm.nih.gov/geo/query/acc.cgi?acc=GSE235449) [71] for the immune cell scRNA-seq dataset. The spatial transcriptome dataset can be accessed with the accession number GSE235315 (https://www.ncbi.nlm.nih.gov/geo/query/acc.cgi?acc=GSE235315) [72]. The Codes used in this manuscript are available at Zenodo (​​https://doi.org/10.5281/zenodo.7016116) [73].

## Abbreviations

scRNA-seq: Single-cell RNA sequencing
PDAC: Pancreatic ductal adenocarcinoma
IPMN: Intraductal papillary mucinous neoplasms
ADM: Acinar-to-ductal metaplasia
CAF: Cancer-associated fibroblasts
myCAF: Myofibroblastic CAF
iCAF: Inflammatory CAF
apCAF: Antigen-presenting CAF
DEG: Differentially expressed genes
EMT: Epithelial-to-mesenchymal transition
GEO: Gene Expression Omnibus
TCGA: The Cancer Genome Atlas
TF: Transcription factor
UMAP: Uniform Manifold Approximation and Projection
MACS: Magnetic-activated cell sorting
