# Supplementary material for: Integrative analysis of spatial and single-cell transcriptome data from human pancreatic cancer reveals an intermediate cancer cell population associated with poor prognosis
Source: Genome Med. 2024 Jan 31;16:20. doi: 10.1186/s13073-024-01287-7 (PMC10832111; doi:10.1186/s13073-024-01287-7)
Supplement: Supplementary file 2 — Additional file 2: Fig. S1. Representative histology of patient samples. Fig. S2. Identification of the epithelial subpopulations in pancreatic cancer. Fig. S3. Identification of the malignant populations in pancreatic cancer epithelial cells. Fig. S4. Deconvolution of the proliferating epithelial subpopulation. Fig. S5. Identification of the fibroblast subpopulations in pancreatic cancer. Fig. S6. Integration of the fibroblast atlas identifies a fibroblast progenitor population in pancreatic cancer. Fig. S7. Identification of Fb_VIT populations. Fig. S8. Deconvolution of the proliferating fibroblast subpopulation. Fig. S9. The composition of cancer cell and CAF subpopulations across patient clusters. Fig. S10. Identification of Fb_COL9A1 populations. Fig. S11. A strategy to identify marker gene sets with prognostic values in PDAC. Fig. S12. Immune cells in human pancreatic cancer tissue. Fig. S13. Immunohistochemistry (IHC) images of the major cancer cell markers. Fig. S14. Correlation between the TF clusters and epithelial subclusters. Fig. S15. Cellular characteristics of the Ep_VGLL1 population. Fig. S16. Reference single-cell transcriptome dataset for spatial deconvolution. Fig. S17. Representative images of spatial deconvolution of human pancreatic cancer. Fig. S18. Marker gene expressions in spatial transcriptome data. [file 13073_2024_1287_MOESM2_ESM.pdf]

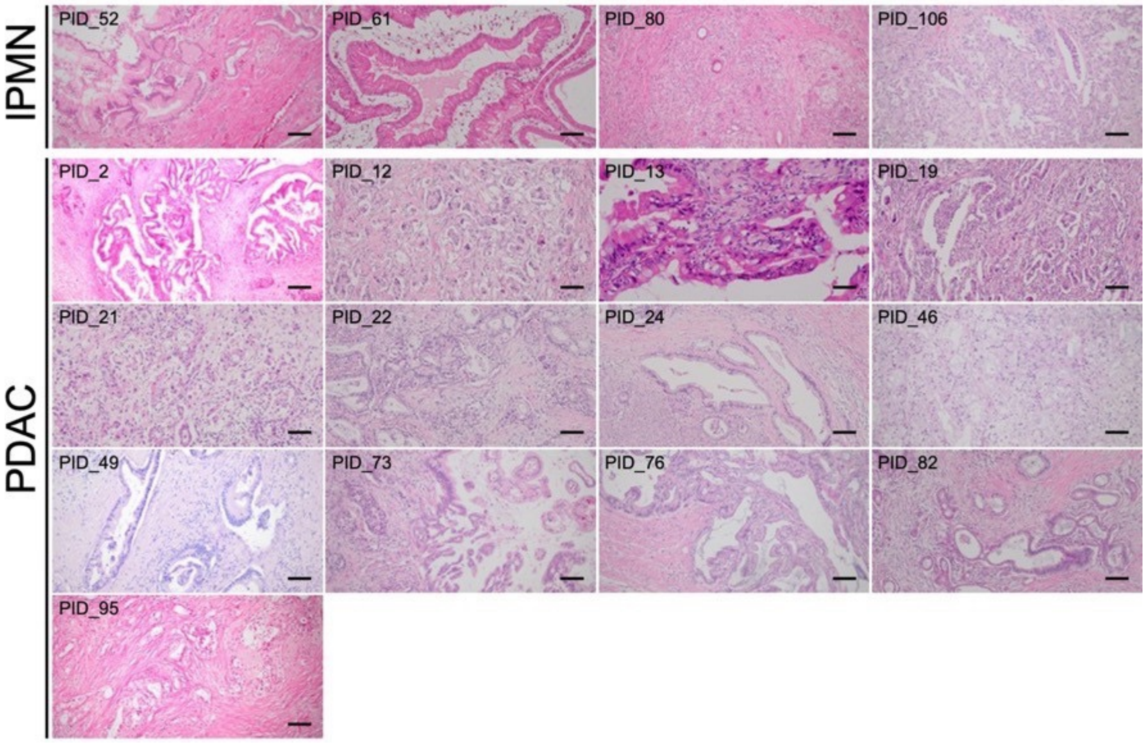

Fig. S1. Representative histology of patient samples. Formalin-fixed paraffin-embedded tissues were sectioned and stained with hematoxylin and eosin (H&E) stain. Representative staining for each patient is shown. All images are x200 magnification images (scale bar=100 µm).

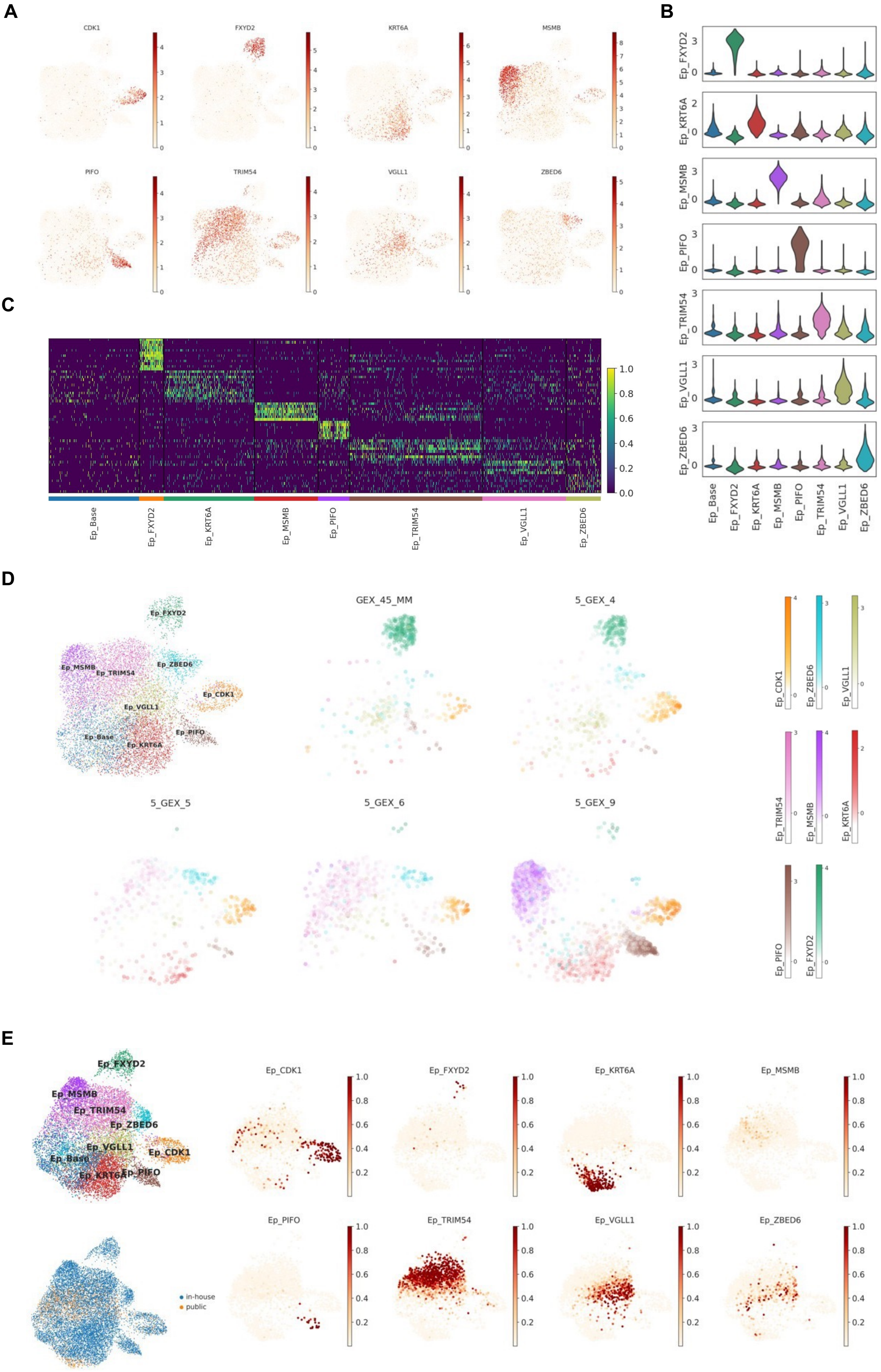
A
B
C
D
E
CDK1
FXYD2
KRT6A
MSMB
PIFO
TRIM54
VGLL1
ZBED6
Ep_FXYD2
Ep_KRT6A
Ep_MSMB
Ep_PIFO
Ep_TRIM54
Ep_VGLL1
Ep_ZBED6
Ep_Base
Ep_CDK1
GEX_45_MM
5_GEX_4
5_GEX_5
5_GEX_6
5_GEX_9
in-house
public

Fig. S2. Identification of the epithelial subpopulations in pancreatic cancer. A, Expression of selected marker genes in total epithelial population. **B**, Violin plots showing the subcluster-specific scores calculated with established marker gene sets for the epithelial population. **C**, Expression of the marker genes are also depicted as a heatmap. Columns in heatmaps consist of epithelial cells grouped by subcluster annotation. **D**, The subcluster-specific scores are projected on each sample comprising the total epithelial population. Only the cells with the upper 10% percentile for each score are shown. **E**, (left) Epithelial cells from a publicly available dataset (Genome Med 2020; 12:80) were merged with the epithelial cell population in our dataset and projected as a UMAP. (right) Prediction probabilities of the epithelial cells from the public dataset are projected on the UMAP. The prediction probabilities were generated from a logistic regression model, which was trained and tested with epithelial cells from this study.

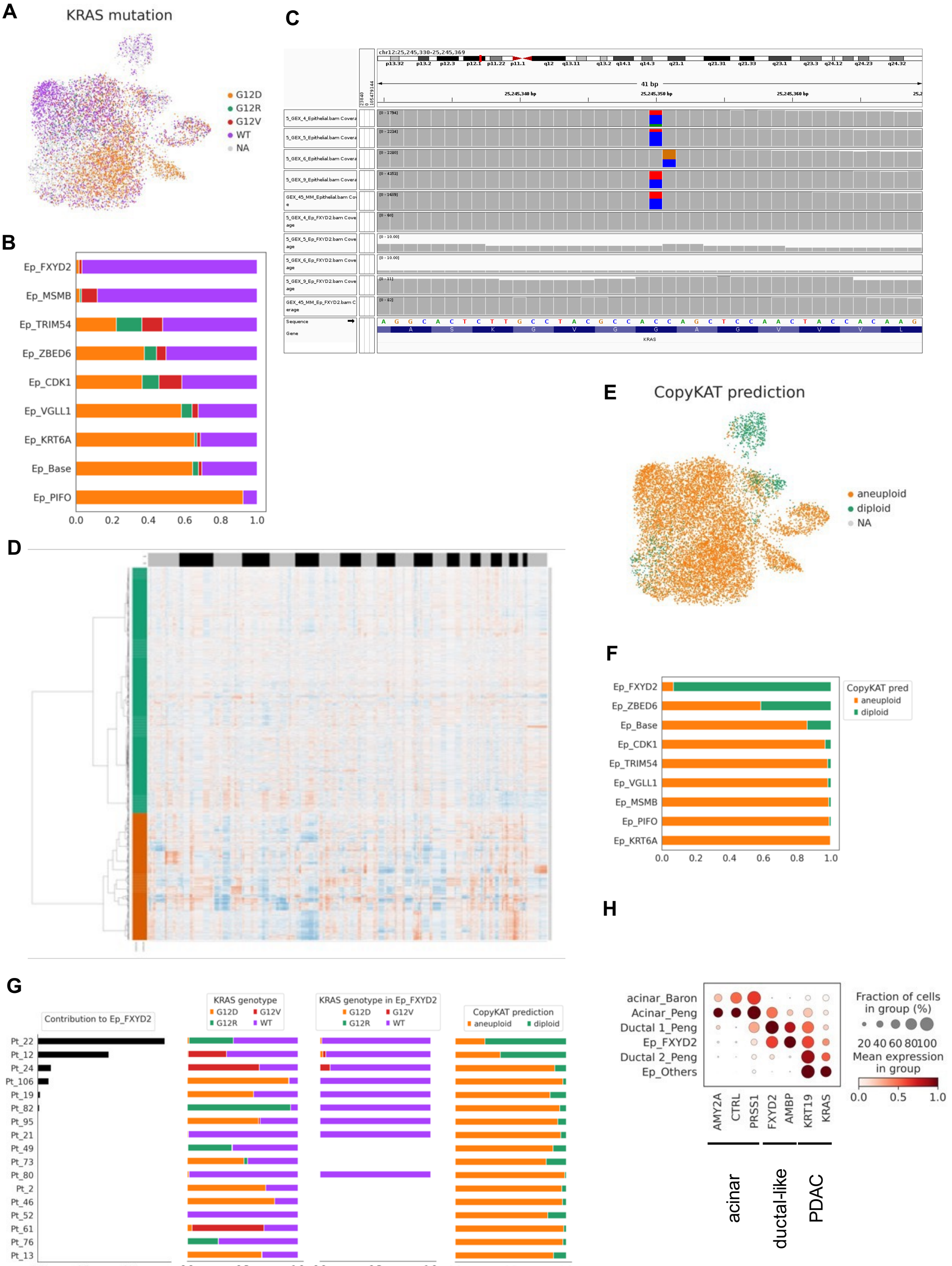
A
KRAS mutation
G12D
G12R
G12V
WT
NA
B
Ep_FXYD2
Ep_MSMB
Ep_TRIM54
Ep_ZBED6
Ep_CDK1
Ep_VGLL1
Ep_KRT6A
Ep_Base
Ep_PIFO
0.0 0.2 0.4 0.6 0.8 1.0
C
KRAS
D
E
CopyKAT prediction
aneuploid
diploid
NA
F
CopyKAT pred
aneuploid
diploid
Ep_FXYD2
Ep_ZBED6
Ep_Base
Ep_CDK1
Ep_TRIM54
Ep_VGLL1
Ep_MSMB
Ep_PIFO
Ep_KRT6A
0.0 0.2 0.4 0.6 0.8 1.0
G
Contribution to Ep_FXYD2
KRAS genotype
G12D G12V G12R WT
KRAS genotype in Ep_FXYD2
G12D G12V G12R WT
CopyKAT prediction
aneuploid diploid
Pt_22
Pt_12
Pt_24
Pt_106
Pt_19
Pt_82
Pt_95
Pt_21
Pt_49
Pt_73
Pt_80
Pt_2
Pt_46
Pt_52
Pt_61
Pt_76
Pt_13
0.0 0.2 0.4
0.0 0.5 1.0
H
acinar_Baron
Acinar_Peng
Ductal 1_Peng
Ep_FXYD2
Ductal 2_Peng
Ep_Others
AMY2A CTRL PRSS1 FXYD2 AMBP KRT19 KRAS
acinar
ductal-like
PDAC
Fraction of cells in group (%)
20 40 60 80100
Mean expression in group
0.0 0.5 1.0

Fig. S3. Identification of the malignant populations in pancreatic cancer epithelial cells. A, UMAP projection of KRAS mutation status in epithelial cells. Mutation status at Gly-12 of KRAS is inferred from the KRAS transcript sequences obtained from each cell. **B**, KRAS mutation status grouped by subcluster annotation and presented as a bar plot. **C**, IGV showing the KRAS mutation status at Gly12 (chr12:25,245,349-25,245,351). Upper five panels represent bam files from the epithelial cells of each sample. Lower five panels show the bam files from the Ep_FXYD2 cluster of each sample. **D**, Heatmap representation of the CopyKAT result. Gray and black boxes in the columns indicate chromosome numbers and locations. Orange boxes in rows indicate aneuploid cells. Green boxes indicate diploid cells inferred from CopyKAT. **E**, UMAP representation of the CopyKAT result. **F**, CopyKAT-inferred ploidies of epithelial cells in each subcluster. **G**, Bar plots showing (left) the batch (patient) composition of the Ep_FXYD2 population, (middle-left) KRAS mutation status of epithelial cells from each patient, (middle-right) KRAS mutation composition in Ep_FXYD2 cells from each patient, (right) and CopyKAT prediction results for epithelial cells from each patient. **H**, Expression of acinar cell, ADM cell and ductal cell markers in pancreatic epithelial cells. 'acinar_Baron' and 'ductal_Baron' cells indicate acinar and ductal cells from normal pancreas tissue (Cell Syst 2016;3(4):346-360), while 'Acinar_Peng', 'Ductal 1_Peng' and 'Ductal 2_Peng' cells indicate acinar and ductal cancer cell populations in pancreatic ductal adenocarcinoma tissues (Cell Res 2019;29(9):725-738). Epithelial cells in this study were split into 'Ep_FXYD2' and 'Ep_Others' as shown in the dot plot.

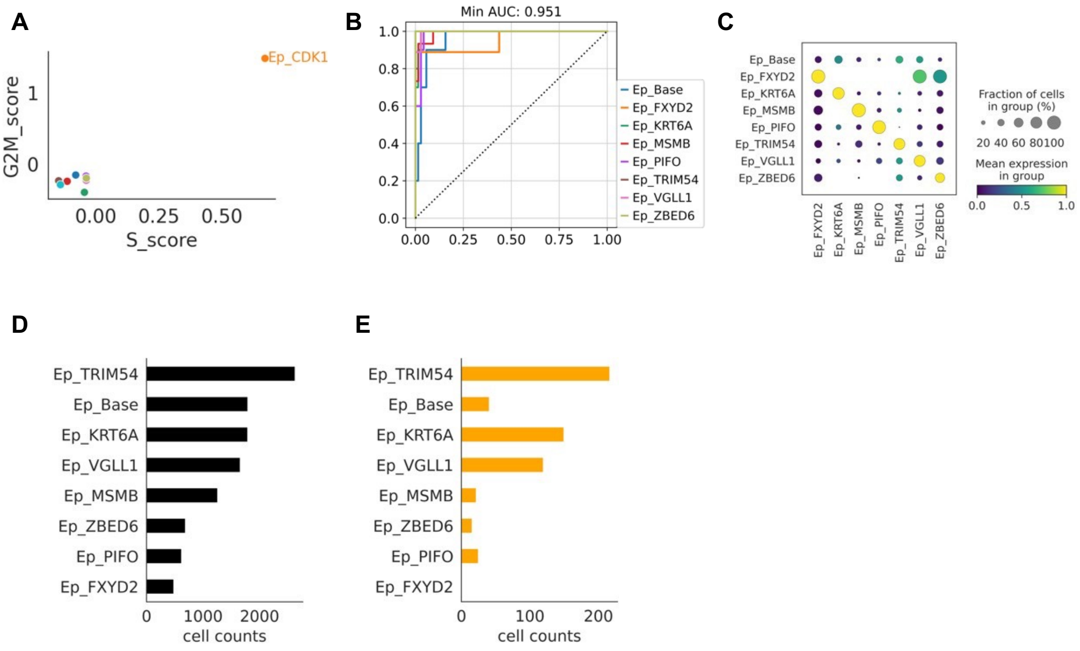

Fig. S4. Deconvolution of the proliferating epithelial subpopulation. A, Scatter plot showing the distinct cycling populations in the epithelial population. The cell cycle state scores were calculated based on phase-specific gene expression. **B**, An ROC curve showing results from the annotation transfer process of the cluster identities. Cluster identities are trained and tested in non-cycling populations and projected to the cycling populations, by a logistic regression method. **C**, Subcluster scores across the subcluster assignment in the Ep_CDK1 population. **D**, Number of epithelial cells in each epithelial subcluster except Ep_CDK1. **E**, Number of Ep_CDK1 cells assigned to each epithelial subcluster.

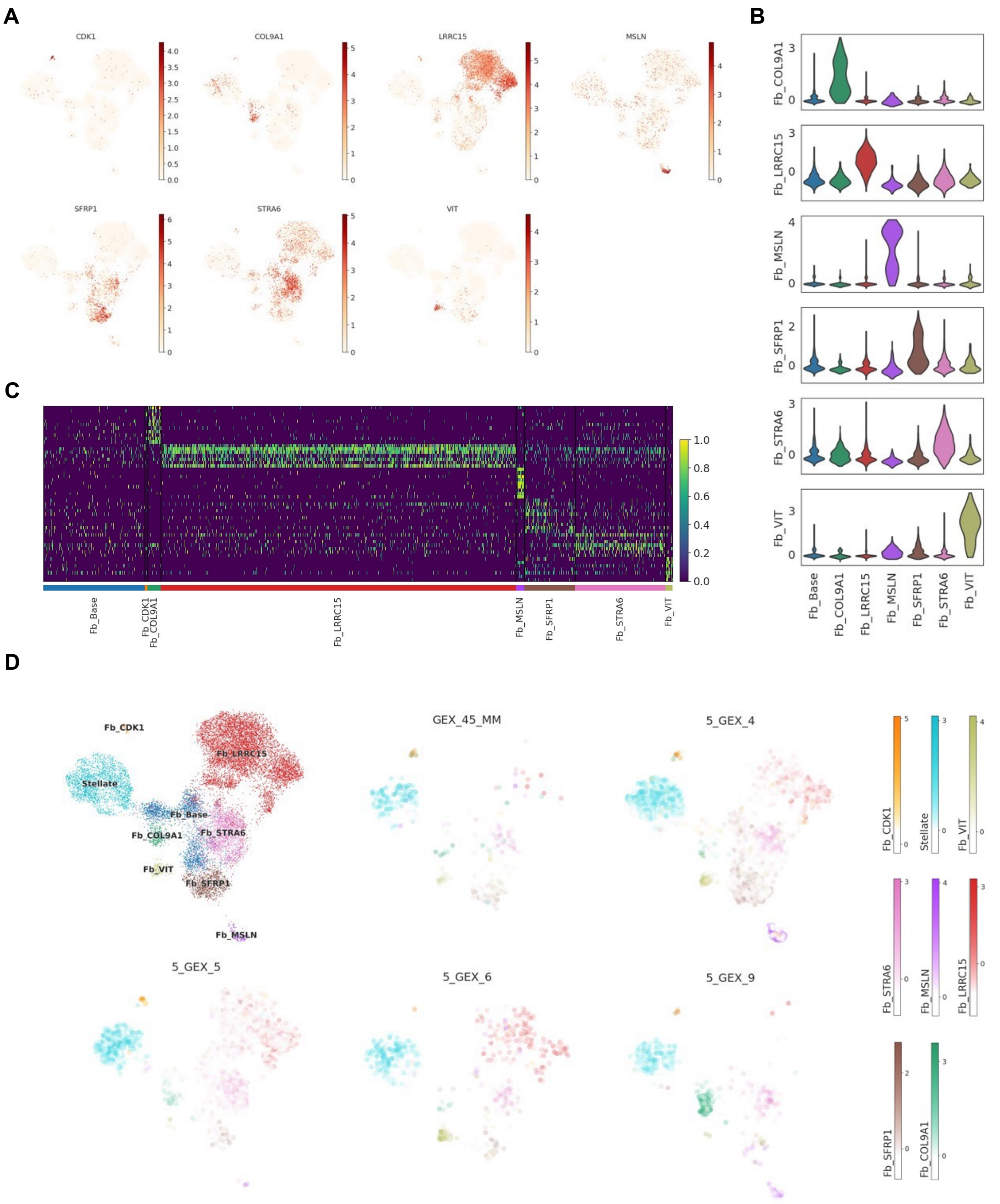

Fig. S5. Identification of the fibroblast subpopulations in pancreatic cancer. A, Expression of selected marker genes in total fibroblast-stellate cell population. **B**, Violin plots showing the subcluster-specific scores calculated with established marker gene sets for the fibroblast population. **C**, Expression of the marker genes are also depicted as a heatmap. Columns in heatmaps consist of fibroblast cells grouped by subcluster annotation. **D**, The subcluster-specific scores are projected on each sample comprising the total fibroblast-stellate cell population. Only the cells with the upper 10% percentile for each score are shown.

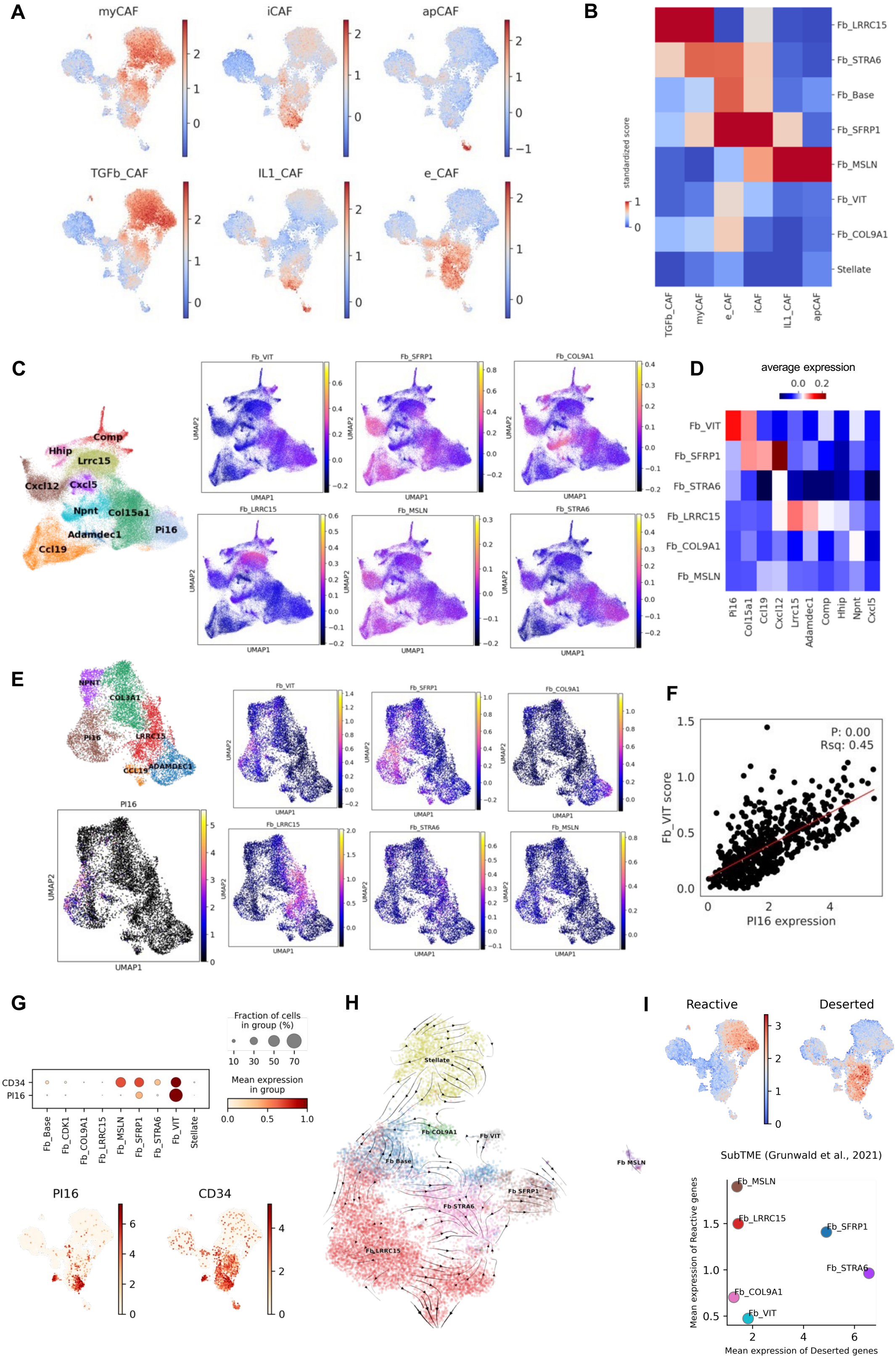
A
myCAF
iCAF
apCAF
TGFb_CAF
IL1_CAF
e_CAF
B
standardized score
Fb_LRRC15
Fb_STRA6
Fb_Base
Fb_SFRP1
Fb_MSLN
Fb_VIT
Fb_COL9A1
Stellate
TGFb_CAF
myCAF
e_CAF
iCAF
IL1_CAF
apCAF
C
Comp
Hhip
Lrrc15
Cxcl12
Cxcl5
Npnt
Col15a1
Pi16
Adamdec1
Ccl19
Fb_VIT
Fb_SFRP1
Fb_COL9A1
Fb_LRRC15
Fb_MSLN
Fb_STRA6
UMAP1
UMAP2
D
average expression
Fb_VIT
Fb_SFRP1
Fb_STRA6
Fb_LRRC15
Fb_COL9A1
Fb_MSLN
Pi16
Col15a1
Ccl19
Cxcl12
Lrrc15
Adamdec1
Comp
Hhip
Npnt
Cxcl5
E
NPNT
COL3A1
PI16
LRRC15
ADAMDEC1
CCL19
PI16
F
P: 0.00
Rsq: 0.45
Fb_VIT score
PI16 expression
G
Fraction of cells in group (%)
Mean expression in group
CD34
PI16
Fb_Base
Fb_CDK1
Fb_COL9A1
Fb_LRRC15
Fb_MSLN
Fb_SFRP1
Fb_STRA6
Fb_VIT
Stellate
PI16
CD34
H
Stellate
Fb COL9A1
Fb VIT
Fb Base
Fb MSLN
Fb SFRP1
Fb STRA6
Fb LRRC15
I
Reactive
Deserted
SubTME (Grunwald et al., 2021)
Fb_MSLN
Fb_LRRC15
Fb_SFRP1
Fb_STRA6
Fb_COL9A1
Fb_VIT
Mean expression of Reactive genes
Mean expression of Deserted genes

Fig. S6. Integration of the fibroblast atlas identifies a fibroblast progenitor population in pancreatic cancer. A, UMAP showing the various CAF subtype scores calculated based on the previously reported gene signatures. **B**, Heatmap representation of CAF scores in fibroblast subclusters. Scores were calculated in each cell based on the average expression of the marker gene sets. The average scores of the cells in each subcluster are standardized and represented as a heatmap. **C**, UMAP representation of the mouse perturbation fibroblast atlas and subcluster scores. Subcluster scores are calculated in the atlas based on the extended marker gene sets of the fibroblast-stellate population. **D**, Average subcluster scores across subcluster annotation. **E**, UMAP representation of the human perturbation fibroblast atlas and calculated subcluster scores. Subcluster scores were calculated in the atlas based on the marker gene sets of the fibroblast population. **F**, Scatter plot showing the correlation of PI16 expression and Fb_VIT score in the human fibroblast perturbation atlas. The P value for the coefficient was determined by a simple linear regression model. **G**, Expression of PI16 and CD34 in the fibroblast-stellate population of pancreatic cancer. **H**, UMAP plot depicting the RNA velocity streamlines. The UMAP projection was obtained from the fibroblast-stellate cell data after regressing out the genes related to proliferation. **I,** Average expression of major DEG (differentially expressed genes) of reactive and deserted TME depicted on the fibroblast UMAP (top) and displayed as a scatter plot (bottom).

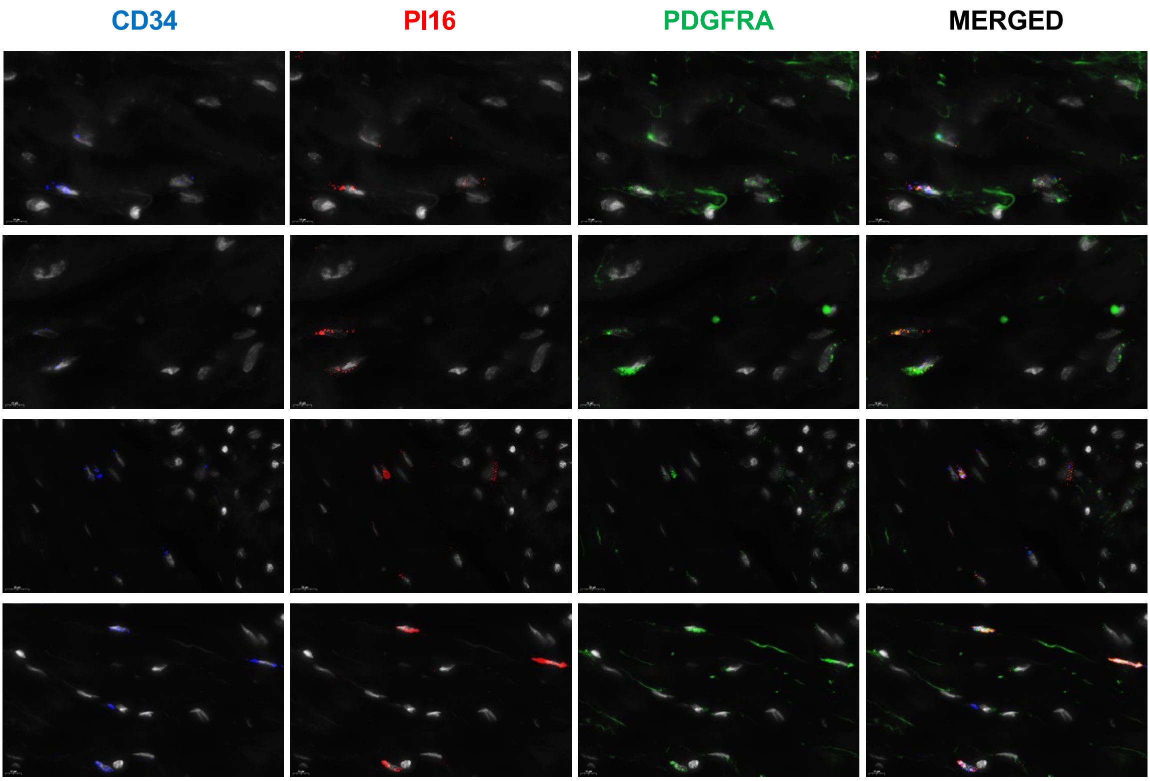

Fig. S7. Identification of Fb_VIT populations. RNA in situ hybridization images of human PDAC tissue. Blue, red and green color indicate CD34, PI16 and PDGFRA, respectively. Scale bars (lower left corner of each image) at the 3rd row indicate 20μm and the scale bars from the rest (1st, 2nd, 4th) of the rows indicate 10μm.

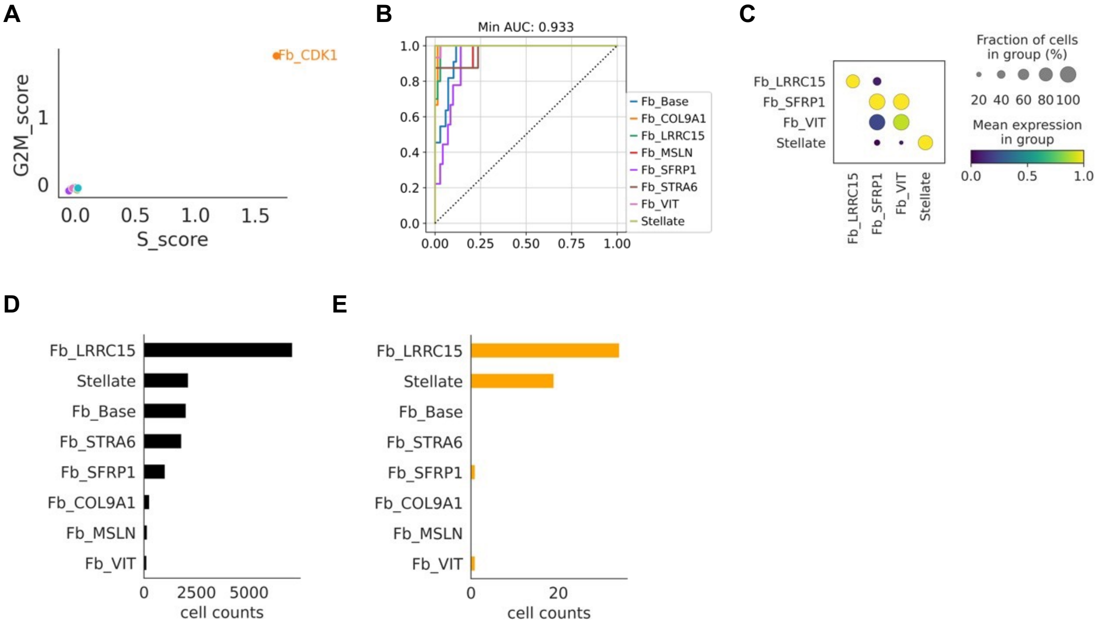

Fig. S8. Deconvolution of the proliferating fibroblast subpopulation. A, Scatter plot showing the distinct cycling populations in the fibroblast population. The cell cycle state scores were calculated based on phase-specific gene expression. **B**, An ROC curve showing results from the annotation transfer process of the cluster identities. Cluster identities are trained and tested in non-cycling populations and projected to the cycling populations, by a logistic regression method. **C**, Subcluster scores across the subcluster assignment in the Fb_CDK1 population. **D**, Number of fibroblasts in each subcluster except Fb_CDK1. **E**, Number of Fb_CDK1 cells assigned to each fibroblast subcluster.

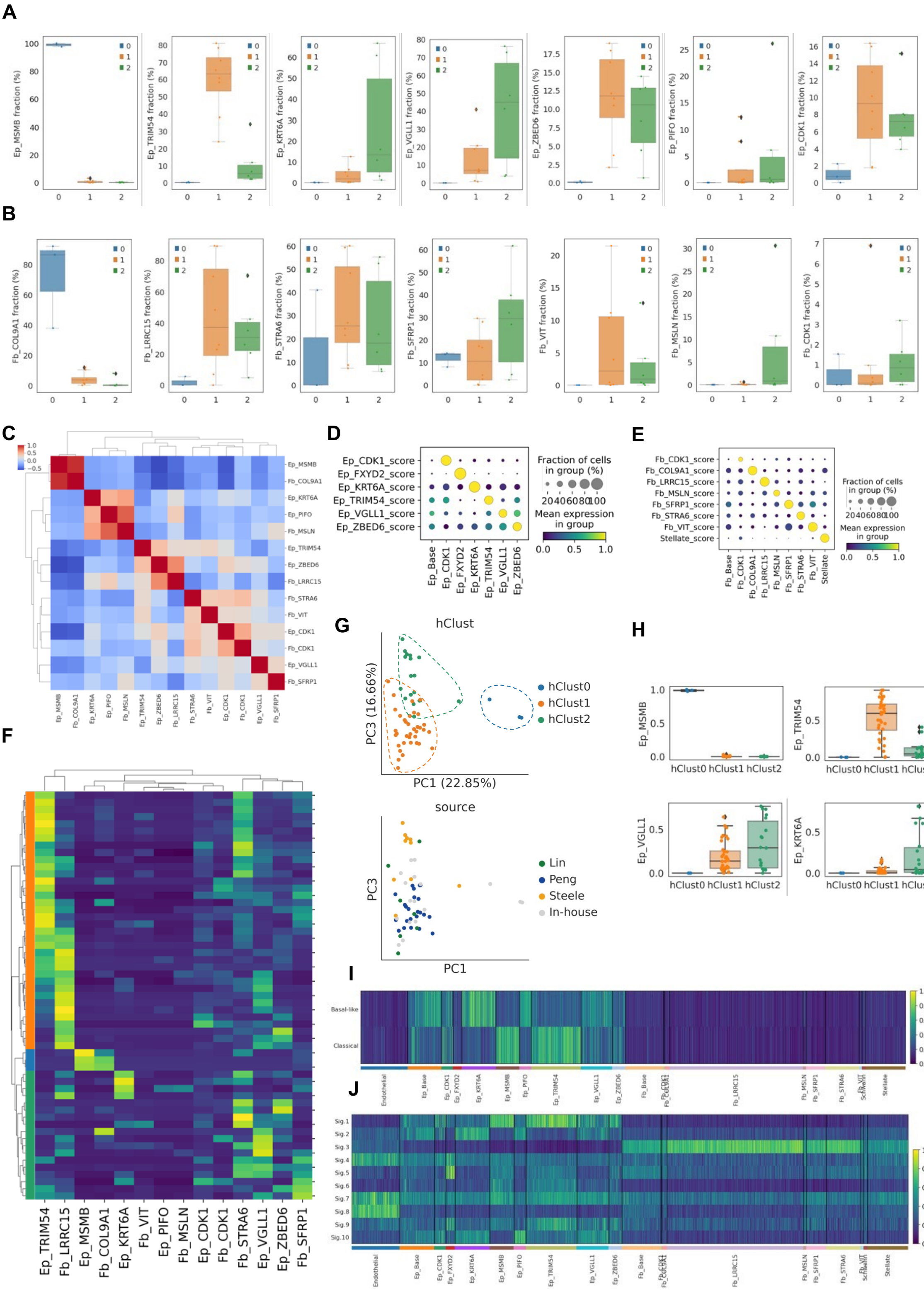
A
Ep_MSMB fraction (%)
Ep_TRIM54 fraction (%)
Ep_KRT6A fraction (%)
Ep_VGLL1 fraction (%)
Ep_ZBED6 fraction (%)
Ep_PIFO fraction (%)
Ep_CDK1 fraction (%)
B
Fb_COL9A1 fraction (%)
Fb_LRRC15 fraction (%)
Fb_STRA6 fraction (%)
Fb_SFRP1 fraction (%)
Fb_VIT fraction (%)
Fb_MSLN fraction (%)
Fb_CDK1 fraction (%)
C
D
Ep_CDK1_score
Ep_FXYD2_score
Ep_KRT6A_score
Ep_TRIM54_score
Ep_VGLL1_score
Ep_ZBED6_score
Fraction of cells in group (%)
Mean expression in group
E
Fb_CDK1_score
Fb_COL9A1_score
Fb_LRRC15_score
Fb_MSLN_score
Fb_SFRP1_score
Fb_STRA6_score
Fb_VIT_score
Stellate_score
F
G
hClust
PC3 (16.66%)
PC1 (22.85%)
hClust0
hClust1
hClust2
source
PC3
PC1
Lin
Peng
Steele
In-house
H
Ep_MSMB
Ep_TRIM54
Ep_VGLL1
Ep_KRT6A
hClust0 hClust1 hClust2
I
Basal-like
Classical
J
Sig.1
Sig.2
Sig.3
Sig.4
Sig.5
Sig.6
Sig.7
Sig.8
Sig.9
Sig.10

Fig. S9. The composition of cancer cell and CAF subpopulations across patient clusters. A, Proportion of each cancer cell subcluster across the patient clusters. **B**, Proportion of each CAF subcluster across the patient clusters. **C**, Heatmap representation of the correlation coefficients of cancer and CAF composition across pancreatic cancer patients. **D-E**, Subcluster scores in **(D)** epithelial subclusters and **(E)** CAF subclusters in public datasets. Cancer cells and CAFs included in public datasets were assigned to each subclusters with logistic regression models, and subcluster scores were calculated by average expression of subcluster-specific marker genes. **F,** Heatmap showing the hierarchical clusters of pancreatic cancer patients in multiple datasets. **G,** PCA representation of the hierarchical clusters. The hierarchical clustering and PCA are based on the composition data of cancer and CAF subpopulations. **H,** Proportions of cancer cell clusters showing differential patterns across the hierarchical clusters. **I**, Expression of basal-like and classical signatures (Nat Genet 2015; 47:1168-78) across the cells from our dataset. **J**, Expression of NMF signature gene sets (Nat Genet 2020; 52:231-40) in PDAC across the cells from our dataset. Whiskers indicate minimum and maximum values, and values exceeding 1.5x IQR (interquartile range) are noted as outliers.

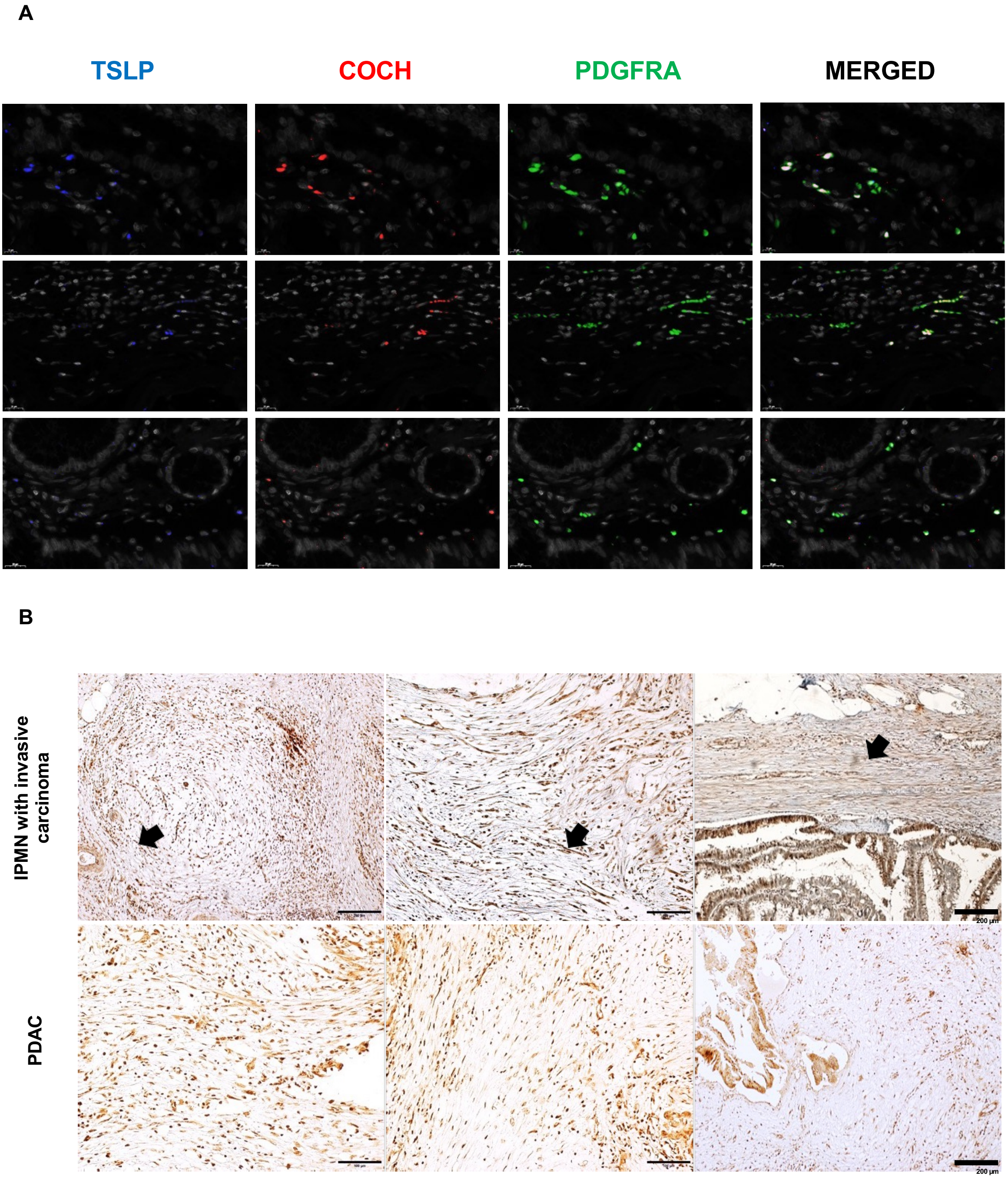

Fig. S10. Identification of Fb_COL9A1 populations. A, RNA in situ hybridization images of human IPMN tissue. Blue, red and green color indicate TSLP, COCH, and PDGFRA, respectively. Scale bars (lower left corner of each image) from the top row indicate 10μm, and the bars in the second and third row indicate 20μm. **B**, Immunohistochemistry (IHC) results. Representative images of anti-COL9A1 (brown) staining in three patients with IPMN (top) and three patients with PDAC (bottom) are shown. Black arrows indicate COL9A1-positive long spindle/stellate cells, morphologically consistent with fibroblasts. Scale bars indicate 200μm.

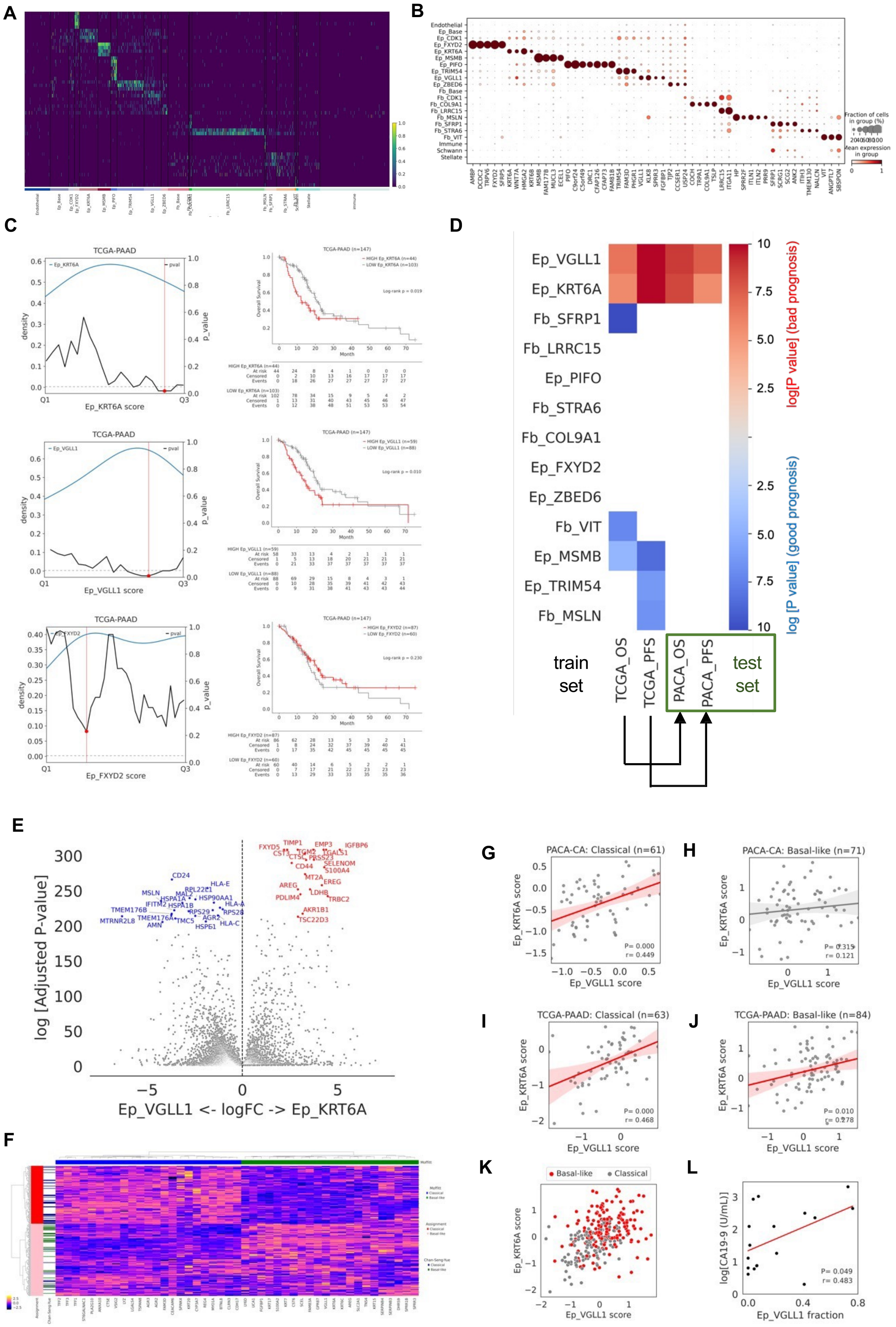
A
B
C
D
E
F
G
H
I
J
K
L
Fraction of cells in group (%)
Mean expression in group
TCGA-PAAD
Ep_KRT6A score
Ep_VGLL1 score
Ep_FXYD2 score
density
p_value
TCGA-PAAD (n=147)
Overall Survival
Month
Log-rank p = 0.019
Log-rank p = 0.010
Log-rank p = 0.230
Ep_VGLL1
Ep_KRT6A
Fb_SFRP1
Fb_LRRC15
Ep_PIFO
Fb_STRA6
Fb_COL9A1
Ep_FXYD2
Ep_ZBED6
Fb_VIT
Ep_MSMB
Ep_TRIM54
Fb_MSLN
log[P value] (bad prognosis)
log [P value] (good prognosis)
train set
TCGA_OS
TCGA_PFS
PACA_OS
PACA_PFS
test set
log [Adjusted P-value]
Ep_VGLL1 <- logFC -> Ep_KRT6A
PACA-CA: Classical (n=61)
PACA-CA: Basal-like (n=71)
TCGA-PAAD: Classical (n=63)
TCGA-PAAD: Basal-like (n=84)
Ep_KRT6A score
Ep_VGLL1 score
P= 0.000
r= 0.449
P= 0.315
r= 0.121
P= 0.000
r= 0.468
P= 0.010
r= 0.278
Basal-like
Classical
log[CA19-9 (U/mL)]
Ep_VGLL1 fraction
P= 0.049
r= 0.483

Fig. S11. A strategy to identify marker gene sets with prognostic values in PDAC. A-B, Expression of genes in the refined marker gene sets represented as (**A**) a heatmap or (**B**) a dot plot. **C**, Examples of the cut-off optimization process. (left) Potential cut-off values in the interquartile boundary of the score distribution were tested and the P values from the log-rank test plotted on the y-axis. The optimized cut-off value (red dots; the cut-off value with the most significant P value) was used to demarcate high and low expression groups. (right) The differential survival of the two groups was plotted in a Kaplan-Meier curve for each marker gene set. **D**, Validation of the optimized cut-off values. Cut-off optimization processes were conducted in TCGA cohort (train set) and applied to either the TCGA cohorts or the PACA cohorts. Patients in each cohort were divided into two groups according to the optimized cutoff values, and the prognostic difference between the two groups were presented as a heatmap according to the log-transformed P values (white: no significances, red: bad prognosis, blue: good prognosis). **E**, Differentially expressed genes between Ep_VGLL1 and Ep_KRT6A. **F**, Assignment of basal-like and classical subtypes using defined signature gene sets. **G-K**, Scatter plot showing the Ep_VGLL1 scores and Ep_KRT6A scores in public PDAC datasets. The correlation of the two scores was tested in (**G**) PACA-CA, classical group; (**H**) PACA-CA, basal-like group; (**I**) TCGA-PAAD, classical group; (**J**) TCGA-PAAD, basal-like group; and in (**K**) the merged cohort data. **L**, Correlation of the logarithmic serum CA 19-9 and the fraction of Ep_VGLL1 in our dataset.

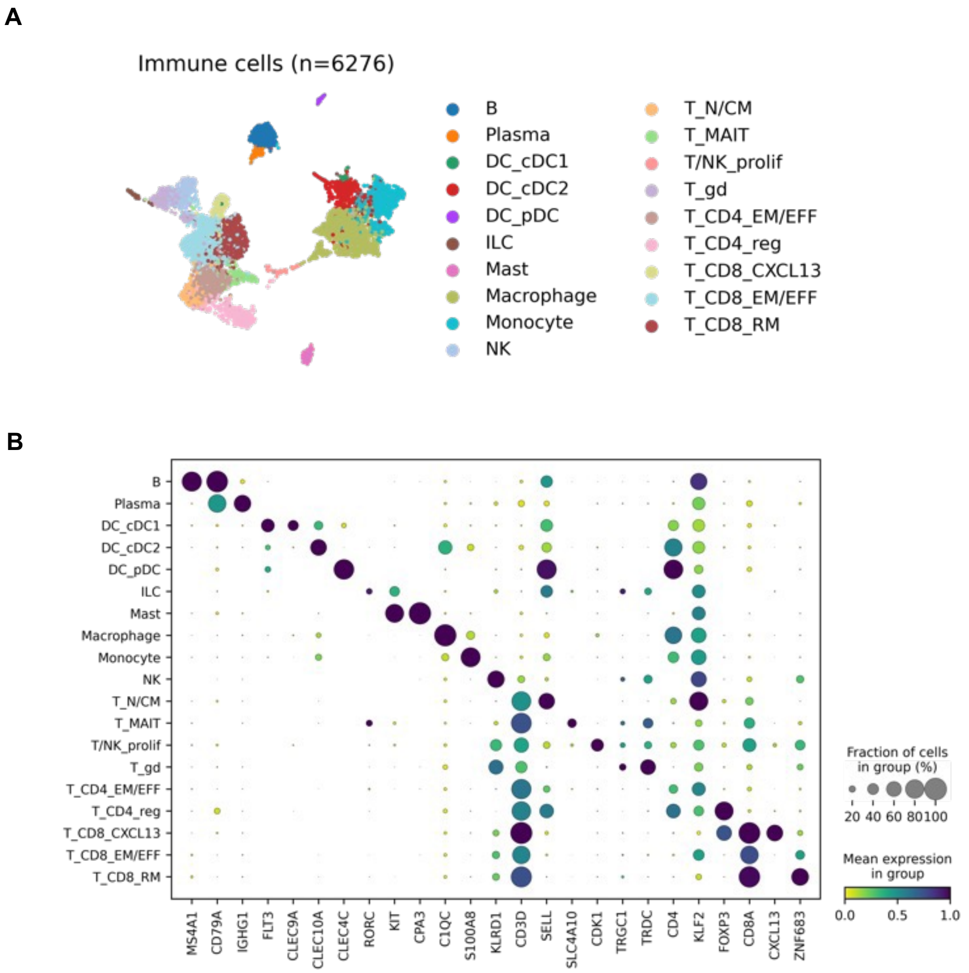

Fig. S12. Immune cells in human pancreatic cancer tissue. A, Landscape of CD45-positive cells projected on UMAP space labeled with cell type annotation. **B**, A dot plot representation of cell type annotations and marker genes.

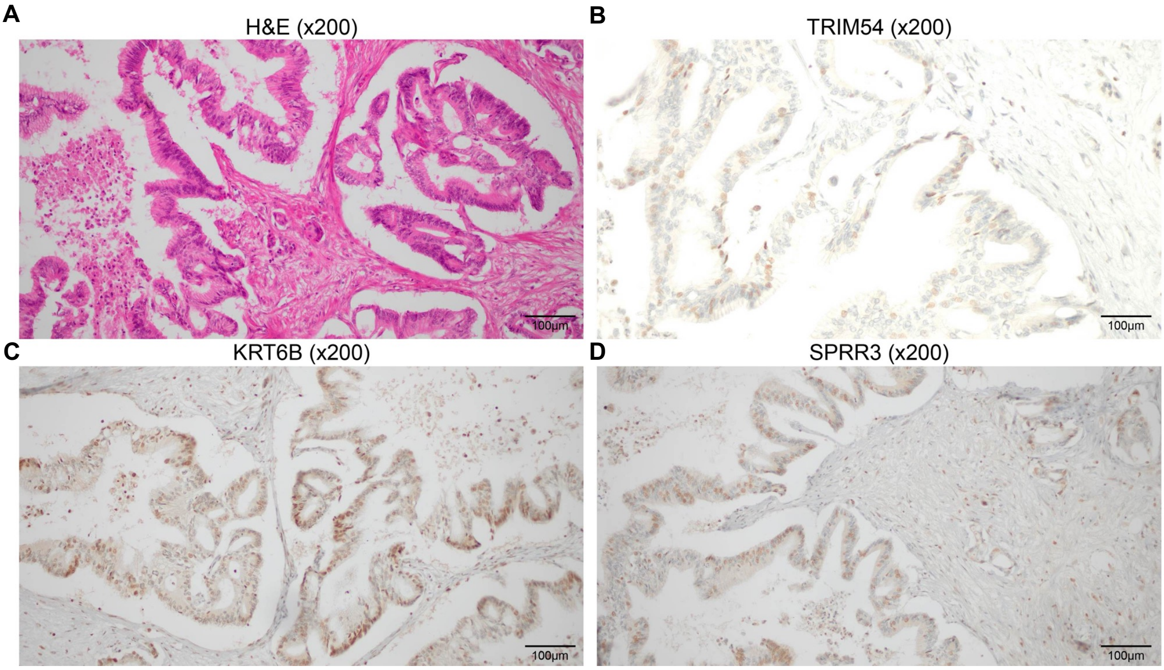

Fig. S13. Immunohistochemistry (IHC) images of the major cancer cell markers. A, Hematoxylin & Eosin (H&E) image of the tissue. **B-D.** Images from IHC assay stained with (**B**) TRIM54, (**C**) KRT6B and (**D**) SPRR3 antibodies, which represents cancer cell clusters of Ep_TRIM54, Ep_KRT6A, and Ep_VGLL1, respectively. Brown colors indicate detection signals from the antibodies.

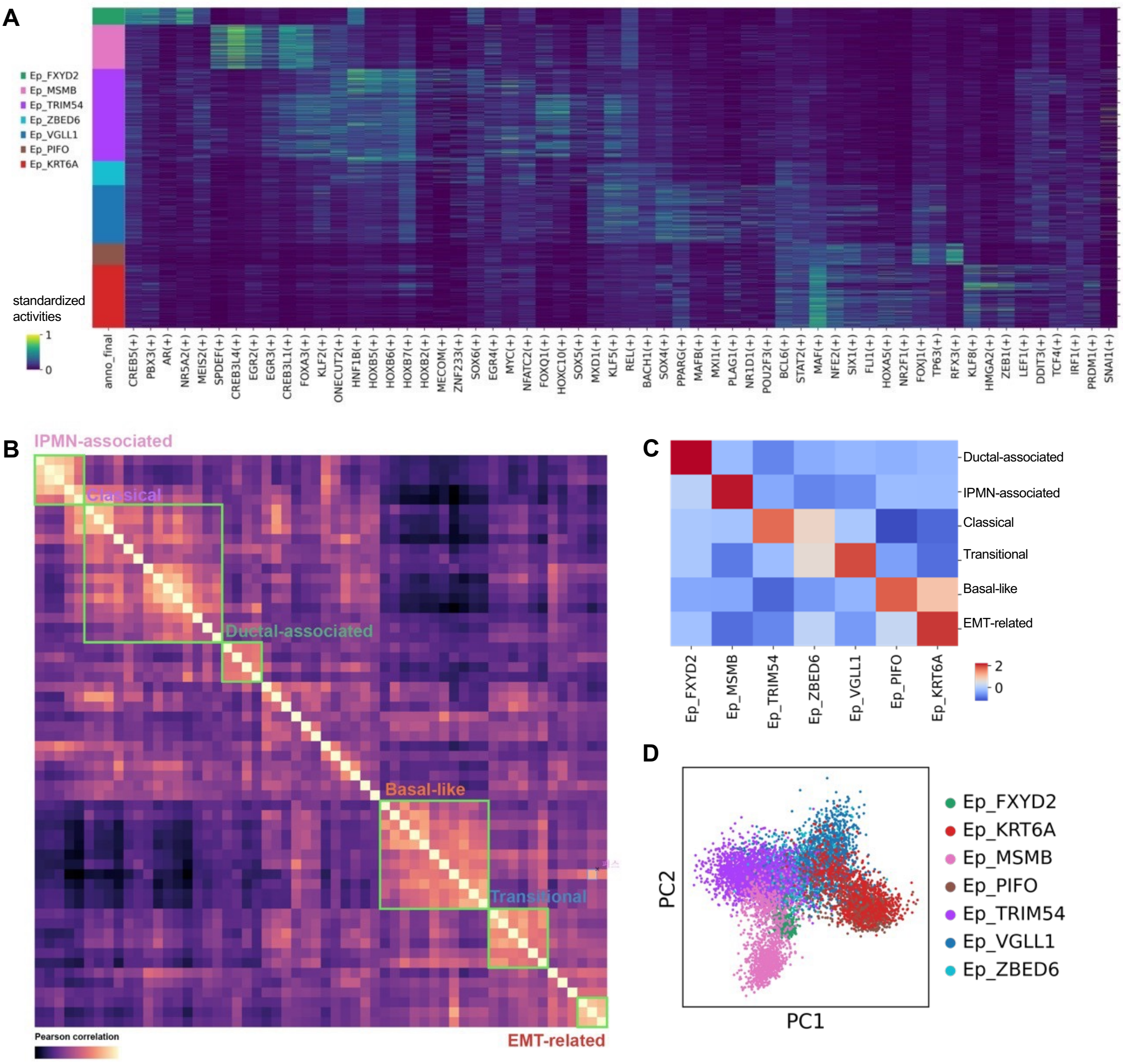

Fig. S14. Correlation between the TF clusters and epithelial subclusters. A, Heatmap showing the inferred TF activities across the cells in each epithelial subcluster. **B**, Correlation matrix of TF activities in pancreatic cancer epithelial cells. Defined TF clusters are shown in green boxes. **C**, Average activities of each TF cluster across the epithelial subclusters. **D**, TF activity-based PCA plot annotated with epithelial cluster identity.

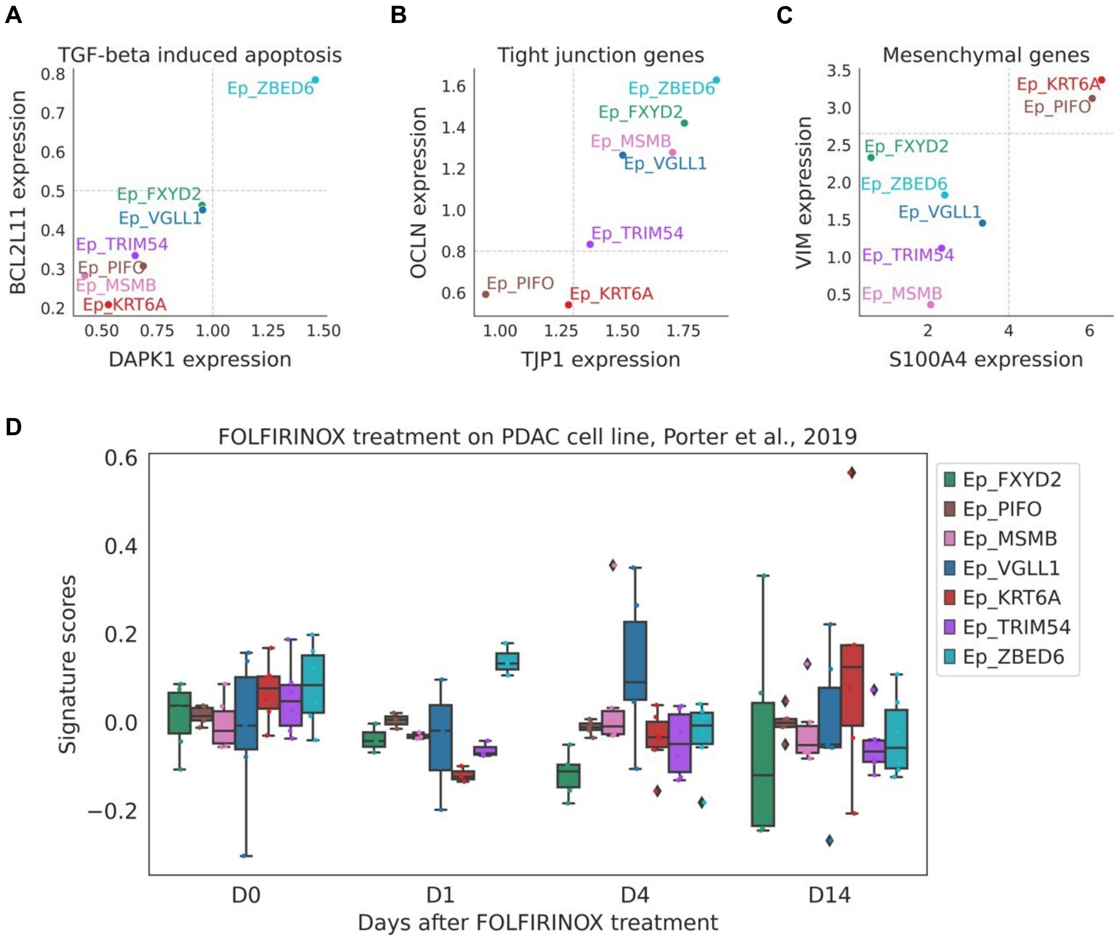

Fig. S15. Cellular characteristics of the Ep_VGLL1 population. A, Average expression of TGF-β-induced apoptosis marker genes, DAPK1 and BCL2L11, across epithelial clusters. **B**, Average expression of tight junction genes across the epithelial clusters. **C**, Average expression of genes representing mesenchymal identities, S100A4 and VIM, across epithelial clusters. **D**, Expression of epithelial cluster marker genes in FOLFIRINOX-treated pancreatic cancer patient-derived organoids. Whiskers indicate minimum and maximum values, and values exceeding 1.5x IQR (interquartile range) are noted as outliers.

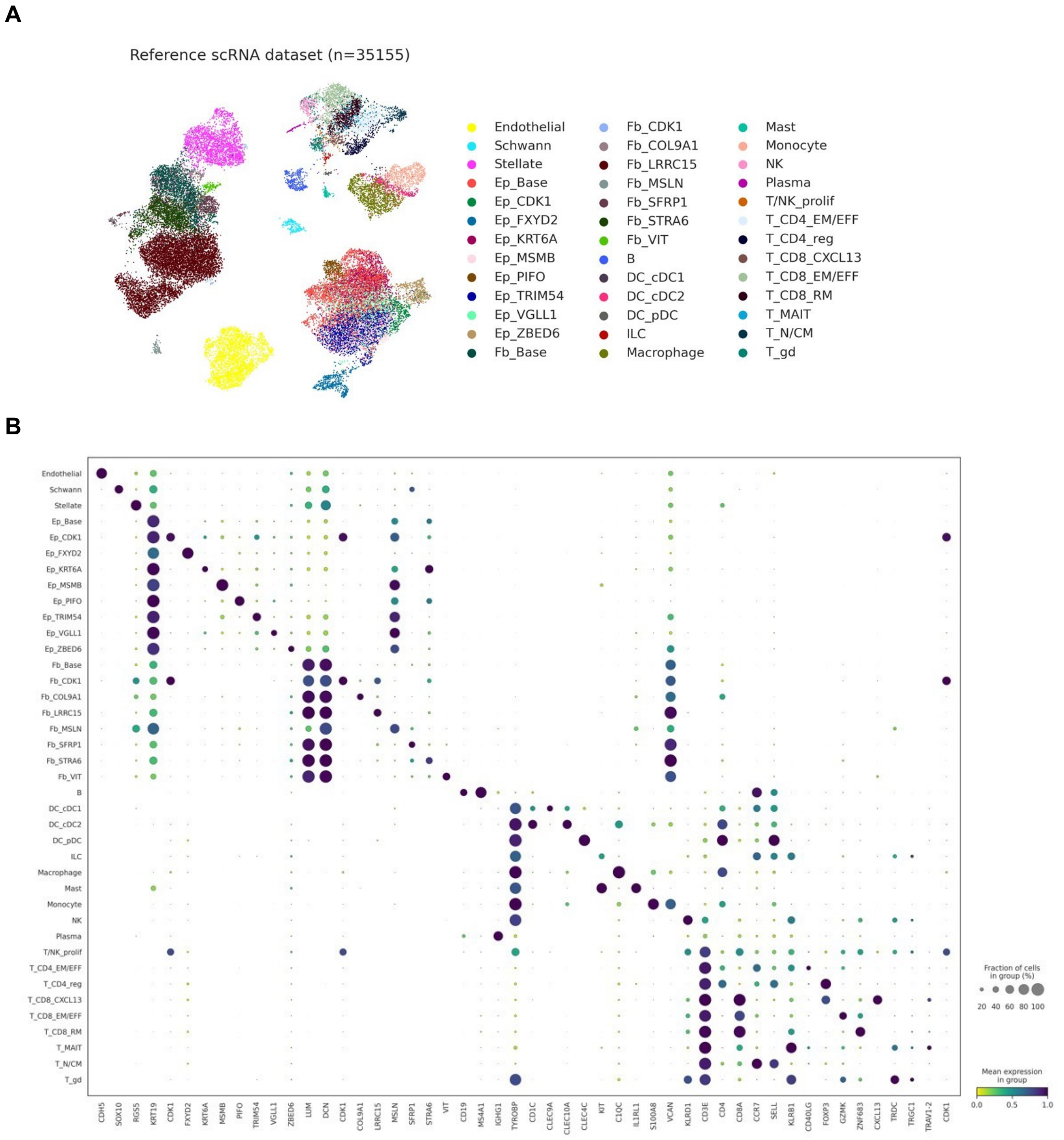

Fig. S16. Reference single-cell transcriptome dataset for spatial deconvolution. A, The integrated single-cell transcriptome data generated in this study is projected on UMAP space. **B**, A dot plot representation of cell type annotations and their marker genes.

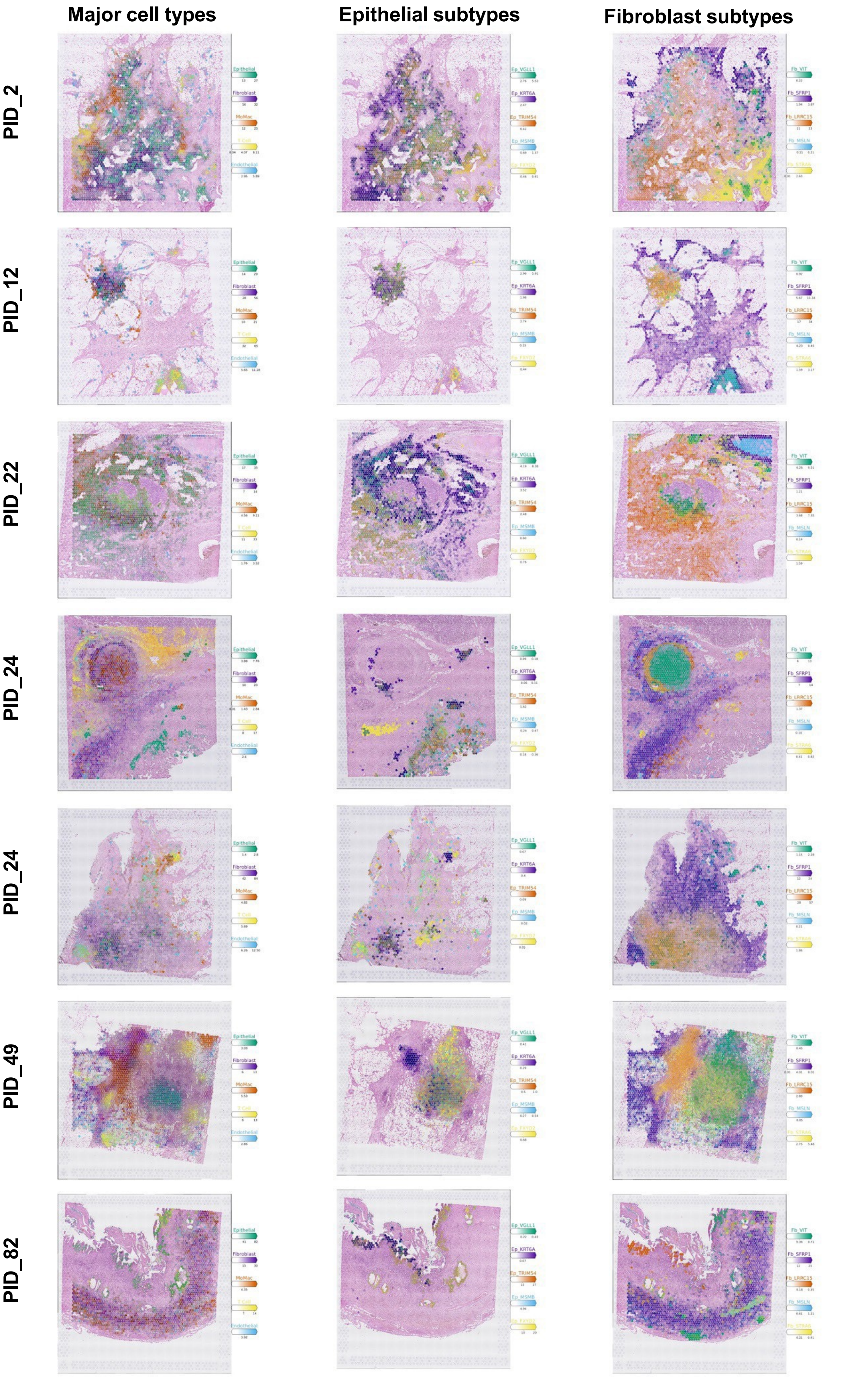
Major cell types
Epithelial subtypes
Fibroblast subtypes
PID_2
PID_12
PID_22
PID_24
PID_24
PID_49
PID_82

Fig. S17. Representative images of spatial deconvolution of human pancreatic cancer. Estimated abundances of the five representative clusters of global cell types(left panel), epithelial subclusters (middle panel) and fibroblast subclusters (right panel) are plotted with different colors for each slide. Estimated abundances and coloring plans are from Cell2location analysis.

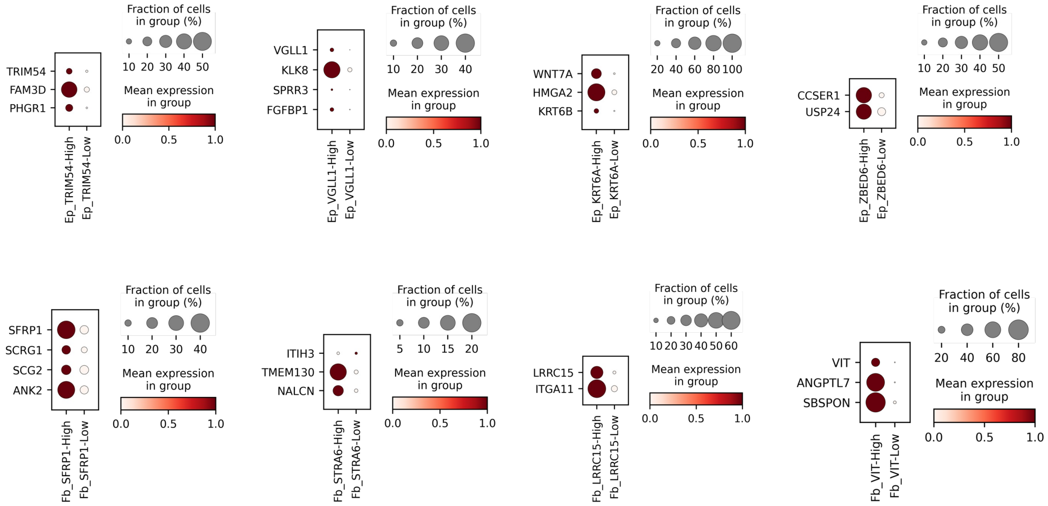

Fig. S18. Marker gene expressions in spatial transcriptome data. Marker genes for epithelial and fibroblast subclusters were compared between cluster-high spots and cluster-low spots. For each epithelial and fibroblast subcluster, cluster- high spots were defined as the spots with estimated abundances over 3, and the rest of the spatial spots were regarded as cluster-low spots. The current version of Visium transcriptomic analysis does not evaluate the full extent of human genes as comprehensively as scRNA-seq analysis; therefore, only the available genes from the marker genes established in scRNA-seq data were evaluated. The subclusters whose number of high-spots were less than 500 were excluded.
