# Supplementary material for: Integrative analysis of spatial and single-cell transcriptome data from human pancreatic cancer reveals an intermediate cancer cell population associated with poor prognosis
Source: Genome Med. 2024 Jan 31;16:20. doi: 10.1186/s13073-024-01287-7 (PMC10832111; doi:10.1186/s13073-024-01287-7)
Supplement: Supplementary file 3 — Additional file 3. Supplementary materials and methods. [file 13073_2024_1287_MOESM3_ESM.docx]

**Supplementary Materials and Methods**

**Single-cell transcriptome data processing**

***Logistic regression for annotation transfer***

We transferred annotation from the reference dataset (single-cell dataset containing reference annotation in AnnData format) to the target dataset using a logistic regression model. To reduce computational complexity, we downsized the reference dataset (i.e., randomly extracted 200 cells in each annotation category and merged them). Next, we randomly split the reference dataset into a train and test set with 4:1 ratio. We trained the model with the LogisticRegression model implemented in the scikit-learn package, using all the genes as features and with ‘l2’ penalty and C=0.2 settings. The performances of the models were evaluated with receiver operating characteristic (ROC) curves. Using the logistic regression model trained from the reference dataset, we generated prediction scores for each entity (i.e., cell) in the reference dataset. We used the prediction scores to annotate the cells in the target dataset, and the predicted annotations were validated with the average expression of the marker genes.

***Identification of cancer cells***

We used CopyKAT (v1.0.4) [[1]](https://paperpile.com/c/DWTKXi/uDhRC) to infer information on copy number alteration. Raw count matrices were merged into a single dataframe and used as the input for CopyKAT. We ran CopyKAT with the default settings, and the prediction result was merged to the processed scRNA-seq dataset for downstream analysis. The KRAS mutation status for each cell was inferred from the reads aligned to chr12:25245349-25245353 (hg38) with pysam (v0.16.0.1). Cells with $\leq$10% of variant allele frequency (VAF) at the target site were annotated as wild-type. Cells with >10% variant sequences were annotated with the most frequent variant sequence.

***Score calculation in the single-cell RNA-sequencing dataset***

Various cell scores were calculated with signature gene sets of interest. Scores were calculated by the average expression of the genes included in the gene set, using the tl.score_genes function implemented in the Scanpy (v1.8.2) package. The signature gene sets were fetched from various sources: The marker gene sets for the CAF subpopulation were used to identify the characteristics of CAF clusters [[2,3]](https://paperpile.com/c/DWTKXi/bMxp+0Mf2), G2M_score and S_score were calculated based on the phase-specific marker gene sets [[4]](https://paperpile.com/c/DWTKXi/B9V6), a list of DEGs between IPMN and PDAC [[5]](https://paperpile.com/c/DWTKXi/UZma) was used to identify the IPMN-related epithelial subcluster, a classifier gene set [[6]](https://paperpile.com/c/DWTKXi/ehGY) was used for basal-like and classical epithelial subclusters, the NMF signature gene sets [[7]](https://paperpile.com/c/DWTKXi/oVpO) were used for signature 1-10 calculation. The EMT score is defined as a mean expression value of the EMT-related genes [[8]](https://paperpile.com/c/DWTKXi/gyMG) and calculated in every individual cell.

***Signaling network analysis***

We used the CellChat [[9]](https://paperpile.com/c/DWTKXi/wLqv) (v1.1.2) package for signaling network analysis according to the instructions provided by the authors.

***Transcription factor activity analysis and diffusion map construction***

We used pySCENIC [[10]](https://paperpile.com/c/DWTKXi/5W33p) (v0.11.2), a python implementation of SCENIC for scRNA-seq data, to infer transcription factor activities within each cell. Non-cycling epithelial cells were analyzed by pySCENIC. All analyses from pySCENIC were carried out with the default parameters provided in the pySCENIC analysis pipeline.

Diffusion map was used to schematize the transcription factor network. We scaled the inferred TF activities from pySCENIC and conducted PCA based on the scaled metrics using pp.scale and tl.pca function in Scanpy package. Next, we computed the neighborhood graph and diffusion map coordinates using pp.neighbors function (n_pcs=10) and tl.diffmap function (n_comps=10) in Scanpy package.

**Population-based clustering**

We first calculated the cancer cell composition and CAF composition in each patient sample and used the proportion data as base metrics. Using the proportion data, we clustered the patients with Ward’s hierarchical clustering method implemented in clustermap function in seaborn package (v0.11.0).

To visualize the patient clusters, we conducted PCA based on the proportion data. We used tl.pca function implemented in Scanpy package. Using the PC coordinates calculated from the package, we plotted the patients on PC axes and labeled them by the cluster identities identified from the hierarchical clustering.

**TCGA Data acquisition**

The RNA-sequencing data from the TCGA-PAAD dataset were downloaded from the PanCanAtlas dataset compilation and PACA-CA data were downloaded from the ICGC browser. Only the samples pathologically annotated as PDAC were included, and the samples with distant metastasis or known previous history of malignancy were excluded.

**REFERENCES**

[1. Gao R, Bai S, Henderson YC, Lin Y, Schalck A, Yan Y, et al. Delineating copy number and clonal substructure in human tumors from single-cell transcriptomes. Nat Biotechnol. Nature Publishing Group; 2021;39:599–608.](http://paperpile.com/b/DWTKXi/uDhRC)

[2. Elyada E, Bolisetty M, Laise P, Flynn WF, Courtois ET, Burkhart RA, et al. Cross-Species Single-Cell Analysis of Pancreatic Ductal Adenocarcinoma Reveals Antigen-Presenting Cancer-Associated Fibroblasts. Cancer Discov. 2019;9:1102–23.](http://paperpile.com/b/DWTKXi/bMxp)

[3. Dominguez CX, Müller S, Keerthivasan S, Koeppen H, Hung J, Gierke S, et al. Single-Cell RNA Sequencing Reveals Stromal Evolution into LRRC15+ Myofibroblasts as a Determinant of Patient Response to Cancer Immunotherapy. Cancer Discov. 2020;10:232–53.](http://paperpile.com/b/DWTKXi/0Mf2)

[4. Tirosh I, Izar B, Prakadan SM, Wadsworth MH 2nd, Treacy D, Trombetta JJ, et al. Dissecting the multicellular ecosystem of metastatic melanoma by single-cell RNA-seq. Science. 2016;352:189–96.](http://paperpile.com/b/DWTKXi/B9V6)

[5. Bernard V, Semaan A, Huang J, San Lucas FA, Mulu FC, Stephens BM, et al. Single-Cell Transcriptomics of Pancreatic Cancer Precursors Demonstrates Epithelial and Microenvironmental Heterogeneity as an Early Event in Neoplastic Progression. Clin Cancer Res. 2019;25:2194–205.](http://paperpile.com/b/DWTKXi/UZma)

[6. Moffitt RA, Marayati R, Flate EL, Volmar KE, Loeza SGH, Hoadley KA, et al. Virtual microdissection identifies distinct tumor- and stroma-specific subtypes of pancreatic ductal adenocarcinoma. Nat Genet. 2015;47:1168–78.](http://paperpile.com/b/DWTKXi/ehGY)

[7. Chan-Seng-Yue M, Kim JC, Wilson GW, Ng K, Figueroa EF, O’Kane GM, et al. Transcription phenotypes of pancreatic cancer are driven by genomic events during tumor evolution. Nat Genet. 2020;52:231–40.](http://paperpile.com/b/DWTKXi/oVpO)

[8. Puram SV, Tirosh I, Parikh AS, Patel AP, Yizhak K, Gillespie S, et al. Single-Cell Transcriptomic Analysis of Primary and Metastatic Tumor Ecosystems in Head and Neck Cancer. Cell. 2017;171:1611–24.e24.](http://paperpile.com/b/DWTKXi/gyMG)

[9. Jin S, Guerrero-Juarez CF, Zhang L, Chang I, Ramos R, Kuan C-H, et al. Inference and analysis of cell-cell communication using CellChat. Nat Commun. 2021;12:1088.](http://paperpile.com/b/DWTKXi/wLqv)

[10. Van de Sande B, Flerin C, Davie K, De Waegeneer M, Hulselmans G, Aibar S, et al. A scalable SCENIC workflow for single-cell gene regulatory network analysis. Nat Protoc. 2020;15:2247–76.](http://paperpile.com/b/DWTKXi/5W33p)
